# Estrogen Receptor Modulators in Viral Infections Such as SARS−CoV−2: Therapeutic Consequences

**DOI:** 10.3390/ijms22126551

**Published:** 2021-06-18

**Authors:** Nikita Abramenko, Fréderic Vellieux, Petra Tesařová, Zdeněk Kejík, Robert Kaplánek, Lukáš Lacina, Barbora Dvořánková, Daniel Rösel, Jan Brábek, Adam Tesař, Milan Jakubek, Karel Smetana

**Affiliations:** 1 BIOCEV, First Faculty of Medicine, Charles University, 252 50 Vestec, Czech Republic; nikita.abramenko@lf1.cuni.cz (N.A.); frederic.vellieux@lf1.cuni.cz (F.V.); zdenek.kejik@lf1.cuni.cz (Z.K.); robert.kaplanek@lf1.cuni.cz (R.K.); lukas.lacina@lf1.cuni.cz (L.L.); barbora.dvorankova@lf1.cuni.cz (B.D.); milan.jakubek@lf1.cuni.cz (M.J.); 2 Department of Paediatrics and Inherited Metabolic Disorders, First Faculty of Medicine, Charles University and General University Hospital, 120 00 Prague, Czech Republic; 3 Department of Oncology, First Faculty of Medicine, Charles University and General University Hospital, 120 00 Prague, Czech Republic; petra.tesarova@vfn.cz; 4 Institute of Anatomy, First Faculty of Medicine, Charles University, 120 00 Prague, Czech Republic; 5 Department of Dermatovenereology, First Faculty of Medicine, Charles University and General University Hospital, 120 00 Prague, Czech Republic; 6 BIOCEV, Faculty of Sciences, Charles University, 252 50 Vestec, Czech Republic; daniel.rosel@natur.cuni.cz (D.R.); jan.brabek@natur.cuni.cz (J.B.); 7 Department of Neurology, First Faculty of Medicine, Charles University, 120 00 Prague, Czech Republic; adam.tesar@vfn.cz

**Keywords:** COVID-19, SARS−CoV−2, estrogen, estrogen receptor, viral replication, IL-6, cytokine storm

## Abstract

COVID-19 is a pandemic respiratory disease caused by the SARS−CoV−2 coronavirus. The worldwide epidemiologic data showed higher mortality in males compared to females, suggesting a hypothesis about the protective effect of estrogens against severe disease progression with the ultimate end being patient’s death. This article summarizes the current knowledge regarding the potential effect of estrogens and other modulators of estrogen receptors on COVID-19. While estrogen receptor activation shows complex effects on the patient’s organism, such as an influence on the cardiovascular/pulmonary/immune system which includes lower production of cytokines responsible for the cytokine storm, the receptor-independent effects directly inhibits viral replication. Furthermore, it inhibits the interaction of IL-6 with its receptor complex. Interestingly, in addition to natural hormones, phytestrogens and even synthetic molecules are able to interact with the estrogen receptor and exhibit some anti-COVID-19 activity. From this point of view, estrogen receptor modulators have the potential to be included in the anti-COVID-19 therapeutic arsenal.

## 1. Introduction

**Figure 1:**
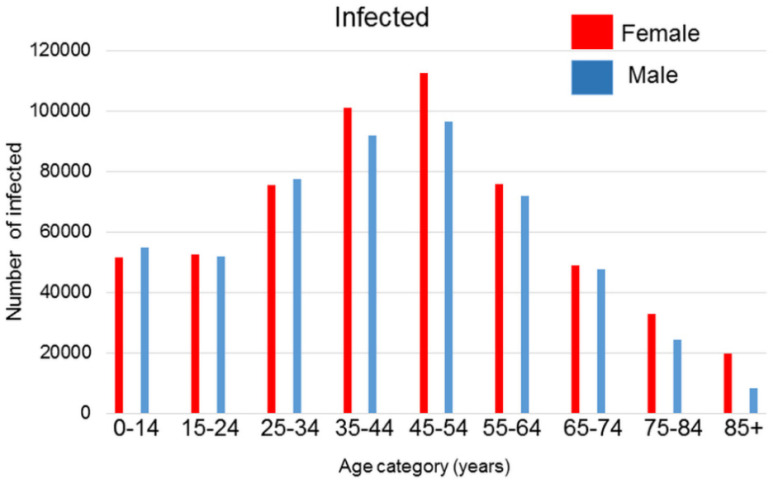
Number of SARS−CoV−2 infections in the Czech Republic until mid-February 2021 according to the data published by the Ministry of Health of the Czech Republic.

**Figure 2:**
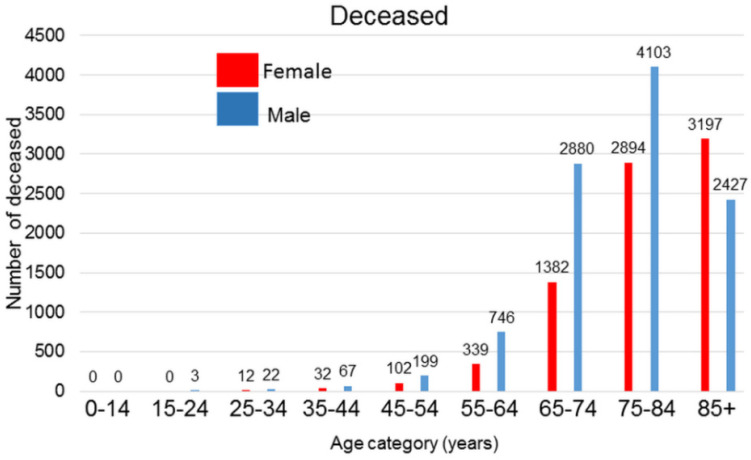
Number of deaths in the Czech Republic until mid–February 2021 according to the data published by the Ministry of Health of the Czech Republic.

COVID-19 is a severe respiratory infection that possesses a pandemic character caused by the SARS−CoV−2 coronavirus, which is a virus similar to the SARS and MERS viruses. Published global epidemiologic data show that while the incidence of infection is only negligibly sex-dependent with some protective role of estrogens in premenopausal women, males die more frequently than females [1,2]. Data from the Ministry of Health of the Czech Republic released up to mid-February 2021 does not contradict these observations (Figure 1 and Figure 2). Experimental data obtained from ovariectomized mice showed more severe courses of SARS−CoV or MERS infections than in control animals [3], which also supports the hypothesis that estrogens possess a certain protective effect against viral infection in females because of their anti-inflammatory and immunomodulatory effect [4]. However, data concerning the role of estrogens are equivocal because there are no significant differences in mortality observed between premenopausal and postmenopausal Chinese women [5]. This can be due to the high dietary intake of isoflavonoids in Eastern Asia.

The interaction of SARS−CoV−2 with its ACE2 receptor (receptor used by the SARS−CoV−2 virus to enter host cells) activates the ADAM17 membrane protease, which can remove ACE2 from the cell membrane, and soluble ACE2 in the serum negatively influences the cardiovascular system [2]. Moreover, ADAM17 also activates TNFα and, through the cleavage of the IL6-receptor, it also stimulates the IL-6 signalling cascade [6,7]. This mechanism seems to be associated with the absence of estrogens and low levels of testosterone in aged males and is likely linked to their unfavourable prognosis following SARS−CoV−2 infection [8]. Thus, the sex-dependent difference and the more severe disease progression resulting in higher mortality of males could also be ascribed to the effect of estrogens in females resulting in lower expression of SARS−CoV−2 receptor [9,10].

On the other hand, the male gender and the presence of androgens could be a predisposing factor for severe COVID-19 cases because androgens appear to ease the entry of the virus into the host cell [11,12]. This observation is supported by an independent study performed in Peru, where male COVID-19 patients with androgenic alopecia suffer from severe COVID-19 more frequently than infected males who do not suffer from this type of alopecia [13]. The protective role of estrogens in COVID-19 is also evident in women suffering from polycystic ovary syndrome. In this condition, with an excess of androgens, women have an increased risk of SARS−CoV−2 compared to the control group [14]. In addition to biological factors, the higher COVID-19 fatality of males is also influenced by behavioural factors such as smoking, alcohol consumption or noncompliance to COVID-19 prevention measures, all of which are generally observed more frequently in males [15].

## 2. Physiological Estrogen Receptor-Dependent Effects and Viral Infection Susceptibility

The difference in susceptibility to viral infection in males and females [16] seems to be dependent on the production of sex hormones in women [17]. These steroids have remarkable effects on a multitude of physiological functions.

The biological functions of estrogens are largely modulated by estrogen receptors known as subtypes alpha (ERα) and beta (ERβ). Estrogen receptors are expressed in numerous cell types of various tissues, including the immune system. During physiological conditions, ERs exist as dimers and are stabilized by the binding of either agonists or antagonists. These receptors reach the cell nucleus and control the transcription of specific target genes by binding to associated DNA regulatory sequences. Estrogen receptors control cells and pathways in the innate and adaptive immune system and regulates immune cell development [18]. Estradiol and ER activity show profound effects on innate immune signaling pathways and myeloid cell development [19].

Moreover, several such hormones may be responsible for the poorer course of COVID-19 disease occurring in males [20]. When focusing on the effect of estradiol on human physiology, many authors have observed that this particular steroid hormone significantly influences the respiratory, cardiovascular and immune systems (summarized by Breithaupt-Fallopa et al. [21] and Pinna [22]. Briefly, estradiol decreases recruitment of neutrophils and local cytokine production and possesses an antioedematous effect in the lungs. Estradiol also reduces blood platelet aggregation and increases the number of lymphocytes. This was confirmed mainly for Th1 and virus-specific CD8 T lymphocytes. Therefore, we observe dysregulated immune functions in COVID-19 infected individuals more frequently in males than in females [23].

**Figure 3:**
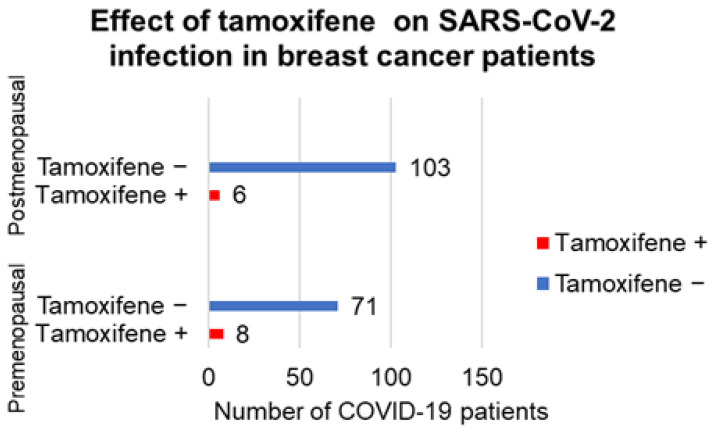
Effect of hormonal therapy of female breast cancer patients with tamoxifene on the sensi-tivity to SARS−CoV−2 infection. Females treated by this drug were not as sensitive to infection as non-treated patients. The effect of therapy was higher in postmenopausal women without the production of estrogens.

On the other hand, tamoxifen is an ERM (estrogen-receptor modulator) that is frequently used to treat breast cancer patients. While short-term tamoxifen application could be helpful in anti-COVID-19 therapy, its long-term application may reduce expression of estrogen receptors (ERs). Hence, this therapy can increase the susceptibility to SARS−CoV−2 [24,25]. In order to address this hypothesis, we retrospectively analysed a group of 273 breast cancer patients suffering from triple-negative and HER2-positive cancer from the Department of Oncology (First Faculty of Medicine, Charles University and General Teaching Hospital, Prague). We focused our analysis on the incidence of clinically manifested infection that was later confirmed in symptomatic patients as COVID-19 by PCR. We reviewed the period during the COVID-19 epidemic, specifically between March 2020 and February 2021, when vaccination became broadly available to our patients. These patients received chemotherapy or endocrine treatment combined with trastuzumab. Patients on immunotherapy, CKD4/6 inhibitors, everolimus or alpelisib were excluded. Similarly, we excluded patients with cancer duplicity or multiplicity and the patients who were already COVID-19 vaccinated. Our data suggest the influence of tamoxifene on SARSe−CoV−2 clinically manifest infection in our breast cancer patients (Figure 3).

From this point of view, males are predisposed to a more severe course of COVID-19 disease [26]. This observation agrees with the results of a genomic investigation using a gene-set enrichment analysis of normal and SARS−CoV−2-infected human tissue, where estrogen receptors ERα and β significantly stimulate the immune response in the infected women. This is in contrast with males, where the immune response is inhibited [27]. Both ERα/β are present in immune cells and their role in COVID-19 may be expected [28,29]. Estrogens stimulate ERα expression in T lymphocytes and activates them [24,30]. The age-dependent loss of ERα in T lymphocytes increases the susceptibility to viruses such is the Coxackie infection [31]. These data may be relevant to the SARS−CoV−2 infection as well.

It should be mentioned here that activities involving the whole-body level function are dependent of the interaction of oestrogens with their specific receptors.

## 3. Effect of Oestrogen Receptor Modulators (ERMs) on Viral Replication

Different modes of action of estrogens and ERMs on viral replication have been described. The mechanism can be either dependent on or independent of the interaction of hormones with their receptors. It has been observed that estradiol interferes with hepatitis B virus infections via the induction of hepatocyte nuclear factor 4α production and interaction with HBV enhancer I [32]. A similar inhibitory effect was confirmed in hepatitis C infections [33,34]. Moreover, these observations are further supported by the evidence of reduced efficiency of hepatitis treatment in postmenopausal women [35]. These mechanisms were also dependent on the interaction of hormones with their receptors. The inhibitory effect of estrogens on replication was documented [36,37,38,39,40,41,42,43,44,45,46,47,48,49,50,51,52,53,54,55,56,57,58,59,60,61,62,63,64,65,66]. in a panel of viruses (for particulars see Table 1).

It seems that estrogens here act independently of their interaction with receptors. What is also noteworthy is that natural and synthetic compounds modulating estrogen receptor activities can be medically relevant. These drugs with ERM activity act as estrogen analogues or agonists (Supplementary Materials Figure S1). However, they can also attenuate the course of viral infection. Unfortunately, the precise molecular mechanisms of action were deciphered only in a few cases and are usually independent of the interaction with the receptor. Clomifene, raloxifene, ridaifene (+XL-147) and toremifene act via attenuation of the interaction of SARS−CoV−2 or EBOLA with their target cells [37,40,41,42,43,44], where some role of the inhibition of SARS−CoV−2 spike protein interaction with receptor ACE2 may be expected [36,37,39,44,45,67]. ERMs seem to inhibit the glycan–glycan interaction between the spike protein and ACE2 and so reduces the virus entry to permissive cells [43,68].

Clomiphene, raloxifene and tamoxifen also interact with late endo/lysosomes. This stimulates accumulation of cholesterol and has an inhibitory effect on the release of SARS−CoV−2 and EBOLA RNA from the vesicle. This represents a critical step in virus replication [39,45,55].

Another compound, genistein, can directly disrupt the replication of viral DNA in the African swine fever virus (ASFV) by interaction with ASFV topoisomerase II [53]. Similarly, quinestrol and raloxifene inhibits the synthesis of SARS−CoV−2 RNA [54].

Cylofenil inhibits assembly and maturation in the Dengue virus [46].

Some candidate drugs can exert several effects in parallel. In addition to targeting virus replication, bazedoxifene can also protect TNF-α-dependent damage to endothelial cells in persons suffering from COVID-19. TNF-α is elevated in this disease as an essential bioactive protein of cytokine storm [68]. Bazedoxifene can block TNF-α binding to CD40 expressed on endothelial cells. This action can prevent endothelial injury [32]. Indeed, epidemiological data suggest that patients on anti-TNF-α drugs for rheumatologic conditions are at lower hospitalisation risk than the control population [69].

These data collectively demonstrate that several ERMs possess remarkable broad antiviral activity independent of their interactions with estrogen receptors. Their activities overlap and can have an inhibitory effect on the viral entry, release of nucleic acid from virions encapsulated by endosomes and replication of nucleic acid and virus assembly, which represent the crucial steps of virus infection.

## 4. ERMs and Estrogens as Inhibitors of SARS−CoV−2 Proteases

Among the SARS−CoV−2 components, the viral proteases such as main protease (M^pro^) and papain-like protease (PL^pro^) represent molecules critically important in viral replication [70,71]. Proteases, therefore, also offer a potential target for antiviral therapy [72]. A plethora of known protease inhibitors (including but not limited to disulfiram, lopinavir/ritonavir, nelfinavir and danoprevir) can be potentially employed in COVID-19 therapy [70].

Remdesivir, an inhibitor of the viral RNA-dependent RNA polymerase, was used as COVID-19 therapeutic. Interestingly, it is also able to interact with SARS−CoV−2 M^pro^ [73].

Some works suggested that ERMs could also be potent inhibitors of SARS−CoV−2 proteases [74], namely isoflavonoids and raloxifene. Among candidate inhibitors of SARS−CoV−2 M^pro^, quercetin and other similar flavonoids were identified [75,76].

**Figure 4:**
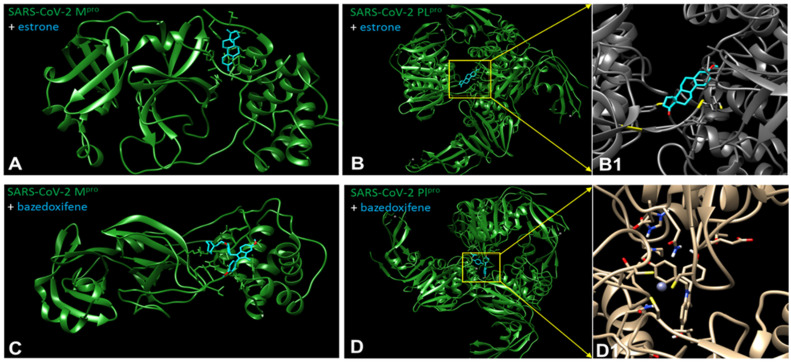
The best docking pose of estrone (above) and bazedoxifene (below) to the SARS−CoV−2 main protease structure (M^pro^) (**A**,**C**) and papain-like protease (PL^pro^) trimer (**B**,**B1**,**D**,**D1**). Pymol representation of the SARS−CoV−2 main protease (PDB id 6y7b) shown as a green ribbon with the secondary structure elements indicated. In blue sticks: the best docking pose for estrone (**A**) or bazedoxifene (**C**). The best docking poses of the estrone (**B**,**B1**) and bazedoxifene (**D**,**D1**) molecule in PL^pro^ trimer (PDB id 6w9c) are localized in the vicinity of the zinc ion present at the central interface of the trimer.

**Figure 5:**
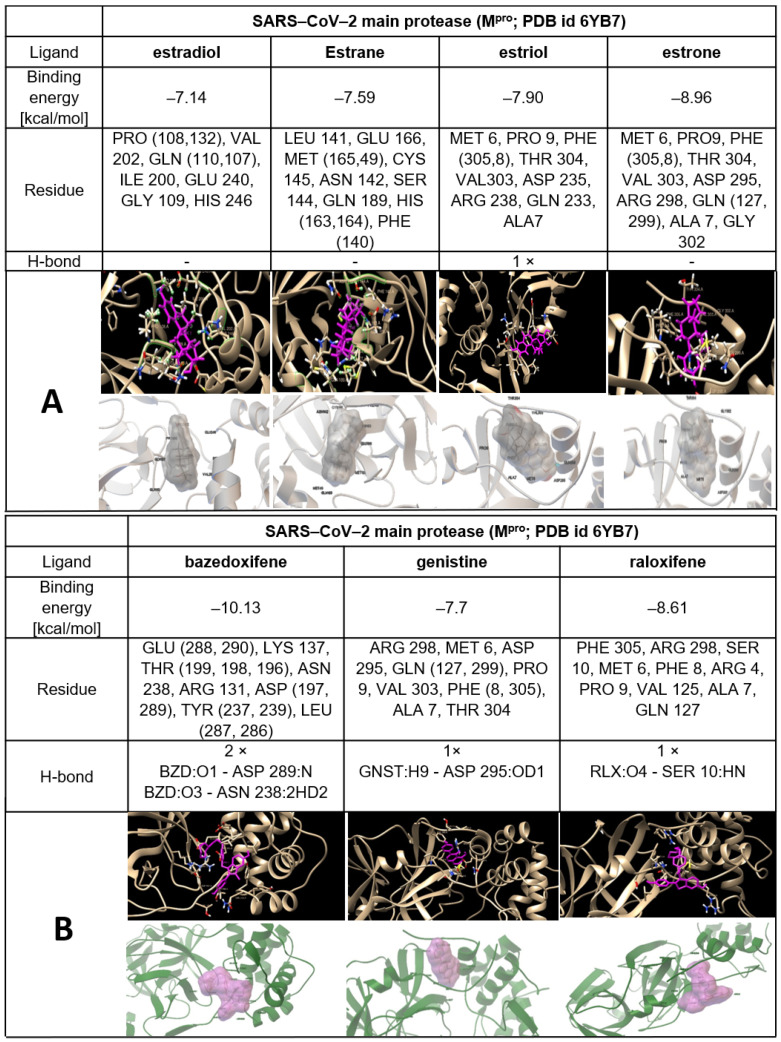
The best docking pose of estrogens (**A**) and other examples of ERM, such as bazedoxifene, genistein and raloxifene (**B**) to the SARS−CoV−2 main protease structure (M^pro^) with characterization of the interaction including hydrogen bonds.

**Figure 6:**
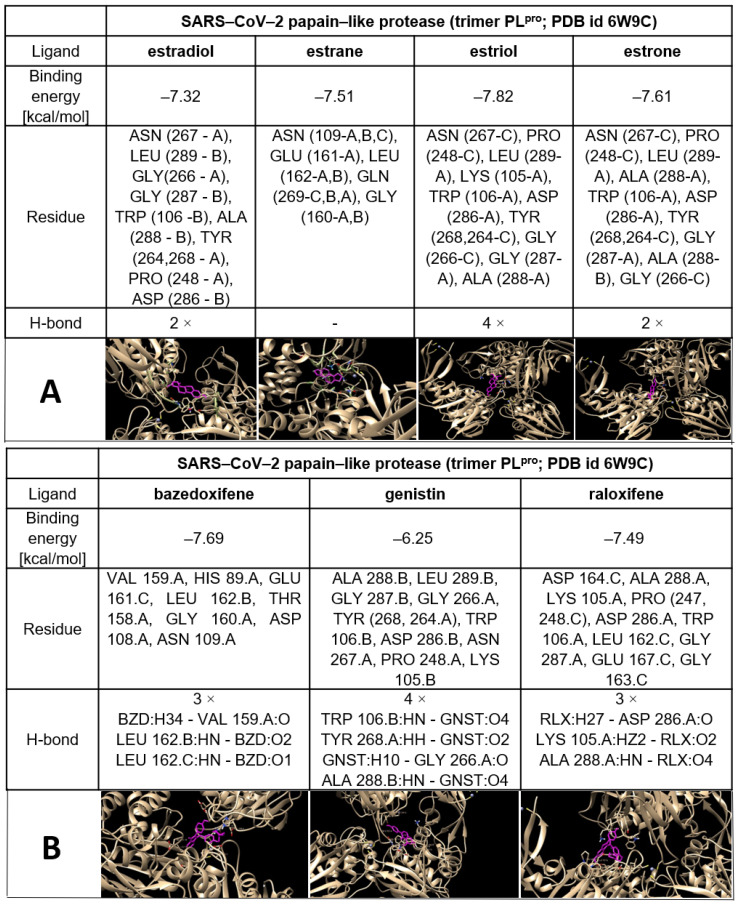
The best docking pose of oestrogens (**A**) and other examples of ERM such as bazedoxifene, genistin and raloxifene (**B**) to the SARS−CoV−2 papain-like protease structure (PL^pro^) (trimer form) with characterization of the interaction including hydrogen bonds.

The possible interactions of ERMs (structures are shown in Supplementary Materials Figure S1) with both SARS−CoV−2 proteases M^pro^ and PL^pro^ are presented in Table 2 and Figure 4, Figure 5 and Figure 6.

Chiou and coworkers [77] have reported that raloxifene is a potent inhibitor of SARS−CoV−2 M^pro^ (IC_50_ = 5.61 μmol/L). It is known that part of the ERM biological effect lies in their interaction with ER. This results in the question of whether estrogens, or the other ERMs could also interact with CoV−2 proteases. In order to address this question, we used AutoDock Vina [78] to predict the binding affinity of genistin, estrogens and ERMS to M^pro^. Based on various interactions, we calculated the binding energy. The docking study implies that ERMs, especially bazedoxifene, display the highest affinity for the SARS−CoV−2 main protease and SARS and the lowest affinity for CoV−2 papain-like protease (PL^pro^ monomer). In the case of estrogens, the values obtained for SARS−CoV−2 M^pro^ were comparable with those calculated for their interaction with PL^pro^ (monomer and trimer) (Table 2, Figure 5 and Figure 6, Supplementary Tables S1–S3 and Figures S2–S22).

These results allow us to postulate the hypothesis that some ERMs, especially bazedoxifene, could display another important therapeutic activity necessary for the treatment of COVID-19 via inhibition of COVID proteases in addition to the inhibition of IL-6 signalling and modulation of ER. The possible inhibitory effect of estrogens against CoV−2 proteases should also be considered when explaining the higher susceptibility of males to COVID-19 than that observed in females. In summary of this section, interaction of ERM including natural estrogens with SARS−CoV−2 proteases can participate in the inhibition of their replication as proposed [72].

## 5. Antiviral Effect of Estrogens as a Developmental Strategy

Sex-specific infection rates and sex-related differences in mortality rates have been documented in humans [79]. Indeed, this is a sort of sexual dimorphism. The hormonal regulation becomes highly dynamic during the course of pregnancy. The development of the hormonal milieu of pregnancy has become an increasingly studied area of interest of immunology in recent years [80]. Pregnancy is associated with an alteration in immune priorities characterised by a strengthening of innate immune barriers and a concomitant reduction in adaptive/inflammatory immunity in the later stages of pregnancy [81]. Briefly, all this precise orchestration of the maternal immune system provides protection for the mother and her future offspring from pathogens, while avoiding detrimental immune responses against the allogeneic foetus. Therefore, estrogen-related signalling seems to be part of a developmental strategy potentially providing critical protection for both the mother and the infant in early life.

The estrogen-ER axis represents a fundamental hormonal regulatory axis of the female reproductive system. This essential regulation influences virtually all body parts in females. This article demonstrates that ER-dependent activity predisposes the organism, namely the respiratory, cardiovascular system and innate/adaptive immunity, to be more resistant to viral infection. Moreover, ER-independent functions can minimise viral entry, replication and assembly.

It is well known in clinics that the female organism in fertile age is more resistant to cardiovascular problems and females have more robust immunity [82]. Klein and Flanagan [16] aimed to summarize the sex-related differences in the immune response of males and females. As confirmed in many species, the immune response is more robust in females than in males (data collected from sea urchin, fruit fly, scorpion fly, wall lizard, Eurasian kestrel, great tit, home mouse, Rhesus macaque and humans). It is also consistent with the fact that women develop a stronger response to vaccination [16].

However, women pay for this advantage by a higher proclivity to autoimmune disorders (e.g., Graves’ disease, Hashimoto thyroiditis and multiple sclerosis).

The activation of ER also stimulates wound healing. It seems to be a critically important aspect of postpartum healing in mammals [83]. However, this impact is broader and also affects extragenital organs. Interestingly, distinct steps of wound healing, such as epidermal cell proliferation or production of extracellular matrix, are precisely regulated by the timely expression of α/β ΕR [84]. What is noteworthy is that the benefits of the female sex are diminished in female mammals after menopause, when the production of sex hormones decreases [16]. Nevertheless, these lost hormone-dependent functions can be restored to some extent by substitutional estrogen therapy and it is used in clinics routinely.

In the context of this article, raloxifene is clinically used as ERM for the prevention of postmenopausal osteoporosis. Moreover, raloxifene administration also normalises levels of blood lipids and thus reduces cardiovascular risks [85].

From the evolutionary view, the summarized plethora of advantages of estrogen during the fertile age can be interpreted as a sort of protection of the female as a donor and bearer of life. Hormonal substitution has had several relevant applications recently and can substantiate therapeutic interventions available in the future. Furthermore, this hypothesis harmonizes well with the lower mortality in women suffering from COVID-19 [86] and may have therapeutic implications.

## 6. Therapeutic Consequences

COVID-19 can be theoretically influenced by the wide panel of approved therapeutics. This repurposing was suggested for angiotensin II receptor blocker (Losartan), protease inhibitors (lopinavir) or biologics inhibiting the IL-6 signalling cascade (Tocilizumab). Corticoids are routinely employed in practice for their broad ability to suppress the immune response [87,88]. Based on extensive knowledge from cancer therapy, inhibitors of sex hormone receptors were also proposed as candidate therapeutics for COVID-19 [25]. As demonstrated in our article, a panel of ERM can be potentially employed [89]. ER of both types are expressed, at the protein level, in cells in both sexes and more in females than in males [90]. However, it is sufficient for clinicians to use estrogen modulators in patients of both sexes. ERM therapy in males is possible for a limited period of time without the adverse effect of feminisation. In addition to the receptor-dependent effect of estrogens controlling the immune response, there is also the direct antiviral effect of ERM that is ER-independent.

Searching for possible off-target indications in approved drugs, ERMs were identified as suitable candidates for COVID-19 therapy [88]. A broad panel of ERMs is routinely used in clinical practice to treat conditions ranging from endocrine disorders to cancer. The combination of ERMs with antiandrogen therapy could also be helpful in the COVID-19 treatment. However, the employment of ERMs in anti-COVID-19 therapy requires further research and clinical testing before their introduction to clinics.

Another strategy can be based on targeting the regulation of androgens. This can result in their temporarily decreased production in COVID-19 patients. This principle was proposed and tested in animal experiments and in a limited clinical study performed in COVID-19 patients [91,92,93].

**Figure 7:**
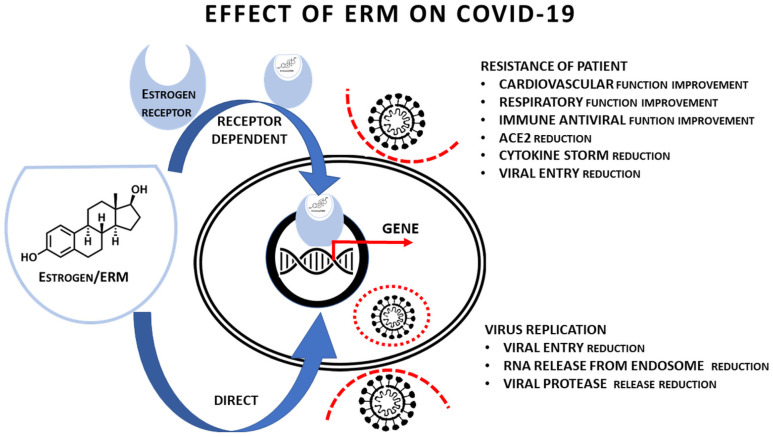
Schematic presentation of the proposed receptor-dependent and independent effect of ERMs on the SARS−CoV−2 infection.

Concerning the hypothesised multiple therapeutic effects of ERMs, bazedoxifene and raloxifene can be good examples. These drugs are very similar with respect to their chemical structures. Both are used for the treatment of postmenopausal osteoporosis [94,95]. As mentioned above, both possess an inhibitory effect on virus replication in which they can interact with viral proteases (Table 2). They also interact with a signal transducer (glycoprotein 130) and, by this mechanism, they attenuate the interaction of IL-6 with its receptor [96,97]. This activity seems to have some anticancer effect. IL-6 is a critical component in the initiation of cytokine storm/hyperinflammatory syndrome that represents the deadly complication of COVID-19 [98,99,100]. Although results are not unambiguous, anti-IL-6 receptor therapy with therapeutic antibody Tocilizumab demonstrated promising effects [101,102,103]. Moreover, bazedoxifene and raloxifene estrogen-like activity and their ability to activate ER may result in beneficial effects on the COVID-19 patients (namely on the lungs, immunity and vascular permeability). The potential effects of ERM on COVID-19 are summarised in Table 3 and in Figure 7, where we demonstrate the effect of ERMs on the organism of infected person and on the virus replication including the effect of ER occupation by suitable ERMs.

The use of ERM, perhaps in combination with other therapeutics, can extend the panel of anti-COVID-19 therapeutics.

## 7. Experimental Part Describing Procedures of Molecular Docking of SARS−CoV−2 Proteases with Estrogens and ERM

For docking studies, the model complexes of SARS−CoV−2 proteases with estrogens and drugs have been created. The three-dimensional structures of proteases were retrieved from the protein data bank database with PDB ID (id 6YB7 for SARS−CoV−2 main protease and id 6W9C for SARS−CoV−2 papain-like protease). The three-dimensional structures of estrogens and drugs with estrogenic effects (Supplementary Figures S2–S22) were obtained from PDB (for estrogens) and PubChem (for drugs) databases. The structures from the PubChem database were saved, using the UCSF Chimera software, as pdb files. During the docking process, one molecule was taken as a receptor and another considered as a ligand and we took a grid box for the receptor. In this study, the protease’s three-dimensional structure was taken as a receptor. The estrogens (estradiol, estrone, estrange and estriol) and ERMs (bazedoxifene, raloxifene and genistein) were considered as ligands. We used AutoDockTools (ADT) in our in silico analysis [78] to prepare receptors and ligands. UCSF Chimera was used to remove two chains from the SARS−CoV−2 papain-like protease (trimeric) to obtain the SARS−CoV−2 papain-like protease (monomeric). Non-essential water molecules, including hetero atoms, were extracted before continuing with the docking test. However, we used ADT for the molecule’s preparation for docking in our analysis. For all our molecules, we used ADT to remove water molecules and solvent residues; we also used a software for adding polar hydrogens and partial chargers to the structure. Molecular docking was finally performed by ADT.

We studied the interaction of proteases with estrogens and ERMs using ADT and UCSF Chimera to identify the protein–ligand binding site. We also tried to define important properties of the new complexes, such as the binding energy between the protein and ligand, the existence of hydrogen bonds and π–π interactions and the presence of amino acids playing a role in the process of binding. The three-dimensional structures were converted to pdbqt format using ADT. The grid box size 126 × 126 × 126 and the centre was selected that covered thes maximum of the surface of the receptor.

After AutoGrid and AutoDock commands we found ten conformations and selected one best based on the power of binding energy and number of H-bonds. The better binding sites (conformations), obtained from the docking process, were found with ADT by function “analysis-conformation-ranked by energy”. After that, we created pdb files of the obtained complexes for the following study of molecules in the UCSF Chimera software [104]. ADT was used to assess the binding affinity of the estrogens to the proteases. In this study, we carried out docking for estradiol, estrone, estrane, estriol, bazedoxifene, raloxifene and genistein. By ADT, we detected information about the binding energy, H-bonds and the existence of π–π interactions. By using the UCSF Chimera software, we obtained information about the binding pockets, such as complete information on what amino acids are involved in the binding process [104]. Moreover, the UCSF Chimera software allowed us to render pictures and label amino acids on them for better visualisation of the binding pockets. Complete information with images is given in separate documents below.

## 8. Conclusions

This paper demonstrates the potential to employ estrogens and modulators of estrogen receptor in COVID-19 therapy.

## Figures and Tables

**Table 1:** Effect of ERMs on the biology of representative viruses.

Substance (Relation to Estrogen)	Virus	Effect
Estradiol	SARS−CoV−2	Blocking of virus entry [36]
Bazedoxifene (agonist/antagonist)	EBOLA SARS−CoV−2	Blocking of endolysosomal system [37] Interaction with SARS−CoV−2 main protease [38]
Clomiphene (analogue)	SARS−CoV−2 EBOLA	Blocking of endolysosomal system [39] Blocking of virus entry [37,40,41,42,43,44,45]
Cyclofenil (agonist/antagonist)	Dengue, Zika	RNA synthesis inhibition [46]
Genestin (analogue)	Adenoviruses Bovine herpesvirus 1 Virus herpes simplex 1/2 Human herpesvirus 8 Moloney murine leukemia Rotaviruses Simian virus 40 Human cytomegalovirus Human immunodeficiency 1	Blocking of virus entry [47] Reduction in virus replication [48] Blocking of virus entry and translation [49] Reduction in virus DNA synthesis [50] Blocking of virus entry [51]
Genistein (analogue)	Arenaviruses Bovine viral diarrhoea African swine fever virus	Inhibition of tyrosinkinase [52] Blocking of virus entry and translation [49] Disruption of DNA synthesis [53]
Quercetin (analogue)	Adenoviruses	Blocking of virus entry and translation [49]
Quinestrol (analogue)	Flaviviruses (ZIKA, Dengue, West Nile)	Reduction in virus RNA synthesis [54]
Raloxifene (agonist/antagonist)	Flaviviruses (ZIKA, Dengue, West Nile) EBOLA, SARS−CoV−2	Reduction in virus RNA synthesis [54,55] Blocking of virus entry [44,56,57,58,59,60]
Ridaifene-b(+XL-147) (Tamoxifen analogue without effect on ER)	EBOLA	Blocking of virus entry [42]
Tamoxifen (agonist/antagonist)	MERS Vesicular stomatitis virus EBOLA	Inhibition of virus replication [61] Inhibition of virus replication and activation of macrophages [62] Blocking of virus entry [45,58]
Toremifene (agonist/antagonist)	MERS EBOLA SARS−CoV−2	Inhibition of virus replication [61] Blocking of virus entry [43,58,63,64] Blocking of virus entry [65,66]

**Table 2:** The calculated interaction energy between SARS−CoV−2 proteases and estrogens or ERMs (kcal/mol).

Agents	Main Protease	Papain-Like Protease (Monomer)	Papain-Like Protease (Trimer)
Estradiol	−7.14	−6.86	−7.32
Estrane	−7.59	−6.28	−7.51
Estriol	−7.9	−6.43	−7.82
Estrone	−8.96	−6.94	−7.61
Bazedoxifene	−10.13	−5.54	−7.69
Genistine	−7.7	−6.07	−6.25
Raloxifene	−8.61	−6.14	−7.49

**Table 3:** Summarization of the potential role of ERMs including estrogen in COVID-19.

Function	Potential Effect of ERM	Dependence on ERM Binding to ER	Potential Clinical/Therapeutic Effect
Pulmonary function	Stimulated	Yes [21,29]	Yes
ACE2 expression in airways	Reduced	Yes [9]	Yes
Cardiovascular function	Stimulated	Yes [21]	Yes
Vascular endothelium injury	Reduced	No [21]	Yes
Increased vascular permeability	Reduced	Yes/No [21]	Yes
Immune response against SARS−CoV−2	Stimulated	Yes [16]	Yes
Sensitivity to vaccination	Stimulated	Yes [16]	Yes
Hypersecretion of cytokines including IL-6	Reduced	Yes [16,68]	Yes
IL-6 binding to receptor	Reduced	No [96,99]	Yes
Virus replication (virus entry)	Reduced	Yes/No (see Table 1)	Yes
Virus replication (release of RNA from endosome)	Reduced	No (see Table 1)	Yes
Virus replication (inhibition of proteases)	Reduced	No (see Table 1, [77])	Yes

## Supplementary Materials

The following are available online at https://www.mdpi.com/article/10.3390/ijms22126551/s1.

## Abbreviations

ACE2	Angiotensin-converting enzyme 2
ADT	AutoDockTools
ER	Estrogen receptor
ERM	Estrogen receptor modulator
HBV	Hepatitis B virus
IL-6	Interleukin-6
M^pro^	Main protease
MERS	Middle East respiratory syndrome
PL^pro^	Papain-like protease
SARS	Severe acute respiratory syndrome
SARS−CoV−2	Severe acute respiratory syndrome-related coronavirus
TNF-α	Tumor necrosis factor-α
