# Supplementary material for: Estrogen Receptor Modulators in Viral Infections Such as SARS−CoV−2: Therapeutic Consequences
Source: Int J Mol Sci. 2021 Jun 18;22(12):6551. doi: 10.3390/ijms22126551 (PMC8233910; doi:10.3390/ijms22126551)
Supplement: Supplementary file 1 [file ijms-22-06551-s001.zip › ijms-1244445-supplementary.pdf]

Supplementary material

Estrogen receptor modulators in viral infections such as SARS-CoV-2: Therapeutic consequences

Nikita Abramenko, Fréderic Vellieux, Petra Tesařová, Zdeněk Kejík, Robert Kaplánek, Lukáš Lacina, Barbora Dvořánková, Daniel Rösel, Jan Brábek, Milan Jakubek and Karel Smetana Jr.

Content

Table S1. Molecular docking of SARS-CoV-2 main protease (M^{pro}) with ERMs
Table S2. Molecular docking of SARS-CoV-2 papain-like protease (mono PL^{pro}) with ERMs
Table S3. Molecular docking of SARS-CoV-2 papain-like protease (trimer PL^{pro}) with ERMs
Figure S1. Structures of ERMs used for docking studies
Figure S2. The best docking pose of estradiol to the SARS-CoV-2 main protease (M^{pro})
Figure S3. The best docking pose of estrane to the SARS-CoV-2 main protease (M^{pro})
Figure S4. The best docking pose of estriol to the SARS-CoV-2 main protease (M^{pro})
Figure S5. The best docking pose of estrone to the SARS-CoV-2 main protease (M^{pro})
Figure S6. The best docking pose of bazedoxifen to the SARS-CoV-2 main protease (M^{pro})
Figure S7. The best docking pose of genistin to the SARS-CoV-2 main protease (M^{pro})
Figure S8. The best docking pose of raloxifene to the SARS-CoV-2 main protease (M^{pro})
Figure S9. The best docking pose of estradiol to the SARS-CoV-2 papain-like protease (mono PL^{pro})
Figure S10. The best docking pose of estrane to the SARS-CoV-2 papain-like protease (mono PL^{pro})
Figure S11. The best docking pose of estriol to the SARS-CoV-2 papain-like protease (mono PL^{pro})
Figure S12. The best docking pose of estrone to the SARS-CoV-2 papain-like protease (mono PL^{pro})
Figure S13. The best docking pose of bazedoxifene to the SARS-CoV-2 papain-like protease (mono PL^{pro})
Figure S14. The best docking pose of genistin to the SARS-CoV-2 papain-like protease (mono PL^{pro})
Figure S15. The best docking pose of raloxifene to the SARS-CoV-2 papain-like protease (mono PL^{pro})
Figure S16. The best docking pose of estradiol to the SARS-CoV-2 papain-like protease (trimer PL^{pro})
Figure S17. The best docking pose of estrane to the SARS-CoV-2 papain-like protease (trimer PL^{pro})
Figure S18. The best docking pose of estriol to the SARS-CoV-2 papain-like protease (trimer PL^{pro})
Figure S19. The best docking pose of estrone to the SARS-CoV-2 papain-like protease (trimer PL^{pro})
Figure S20. The best docking pose of bazedoxifene to the SARS-CoV-2 papain-like protease (trimer PL^{pro})
Figure S21. The best docking pose of genistin to the SARS-CoV-2 papain-like protease (trimer PL^{pro})
Figure S22. The best docking pose of raloxifene to the SARS-CoV-2 papain-like protease (trimer PL^{pro})

Table S1. Molecular docking of SARS-CoV-2 main protease (M^{pro}) with ERMs

	SARS-CoV-2 main protease (M^{pro}; PDB id 6YB7)						
ERM	estradiol	estrane	estriol	estrone	bazedoxifene	genistin	raloxifene
Binding energy [kcal/mol]	-7.14	-7.59	-7.90	-8.96	-10.13	-7.70	-8.61
Residue	PRO (108,132), VAL 202, GLN (110,107), ILE 200, GLU 240, GLY 109, HIS 246	LEU 141, GLU 166, MET (165,49), CYS 145, ASN 142, SER 144, GLN 189, HIS (163,164), PHE (140)	MET 6, PRO 9, PHE (305,8), THR 304, VAL 303, ASP 235, ARG 238, GLN 233, ALA 7	MET 6, PRO 9, PHE (305,8), THR 304, VAL 303, ASP 295, ARG 298, GLN (127, 299), ALA 7, GLY 302	GLU (288, 290), LYS 137, THR (199, 198, 196), ASN 238, ARG 131, ASP (197, 289), TYR (237, 239), LEU (287, 286)	ARG 298, MET 6, ASP 295, GLN (127, 299), PRO 9, VAL 303, PHE (8, 305), ALA 7, THR 304	PHE 305, ARG 298, SER 10, MET 6, PHE 8, ARG 4, PRO 9, VAL 125, ALA 7, GLN 127
H-bond	-	-	1 ×	-	2 × BZD:O1 - ASP 289:N BZD:O3 - ASN 238:2HD2	1 × GNST:H9 - ASP 295:OD1	1 × RLX:O4 - SER 10:HN

Abbreviations: BZD = bazedoxifene; GNST = genistin; RLX = raloxifene

Table S2. Molecular docking of SARS-CoV-2 papain-like protease (mono PL^{pro}) with ERMs

	SARS-CoV-2 papain-like protease (mono PL^{pro}; PDB id 6W9C)						
ERM	estradiol	estrane	estriol	estrone	bazedoxifene	genistin	raloxifene
Binding energy [kcal/mol]	-6.86	-6.28	-6.43	-6.94	-5.54	-6.07	-6.14
Residue	ASN (308,177), SER (239,180), GLU (238,124), PRO 240, CYS 181, LEU (125,178), LYS 126	ASP (164,302), SER 245, TYR (273,264), PRO (247,248), THR 301, MET 208, ARG 166, ALA 246	LYS (217,306), TYR (305,213), THR (257,259), GLU 214, GLY 256, PHE 258	ASN (308,177), SER (239,180), GLU (238,124), PRO 240, CYS 181, LEU (125,178), LYS 126	ASP 134, ASN 177, ILE 14, LYS 126, PHE 127, GLU 70, PRO 130, TYR 71, ASN 128, HIS 73	TYR 95, LYS (91, 94), GLY 142, PRO 96, GLU 143, TRP 93, ALA (145, 144)	LEU (125, 178), SER (180, 239), ASN (177, 308), LYS 126, CYS 181, GLU (238, 124), PRO 240, ASP 179
H-bond	2 ×	-	1 ×	2 ×	2 × BZD:H34 - GLU 70:OE1 BZD:H33 - ASP 134:OD2	1 × GNST:H9 - LYS 91:O	-

Abbreviations: BZD = bazedoxifene; GNST = genistin; RLX = raloxifene

Table S3. Molecular docking of SARS-CoV-2 papain-like protease (trimer PL^{pro}) with ERMs

	SARS-CoV-2 papain-like protease (trimer PL^{pro}; PDB id 6W9C)						
ERM	estradiol	estrane	estriol	estrone	bazedoxifene	genistin	raloxifene
Binding energy [kcal/mol]	-7.32	-7.51	-7.82	-7.61	-7.69	-6.25	-7.49
Residue	ASN 267-A, LEU 289-B, GLY 266-A, GLY 287-B, TRP 106-B, ALA 288-B, TYR (264,268-A), PRO 248-A, ASP 286-B	ASN 109-A,B,C, GLU 161-A, LEU 162-A,B, GLN 269-C,B,A, GLY 160-A,B	ASN 267-C, PRO 248-C, LEU 289-A, LYS 105-A, TRP 106-A, ASP 286-A, TYR (268,264-C), GLY 266-C, GLY 287-A, ALA 288-A	ASN 267-C, PRO 248-C, LEU 289-A, ALA 288-A, TRP 106-A, ASP 286-A, TYR (268,264-C), GLY 287-A, ALA 288-B, GLY 266-C	VAL 159-A, HIS 89-A, GLU 161-C, LEU 162-B, THR 158-A, GLY 160-A, ASP 108-A, ASN 109-A	ALA 288-B, LEU 289-B, GLY 287-B, GLY 266-A, TYR (268, 264-A), TRP 106-B, ASP 286-B, ASN 267-A, PRO 248-A, LYS 105-B	ASP 164-C, ALA 288-A, LYS 105-A, PRO (247, 248-C), ASP 286-A, TRP 106-A, LEU 162-C, GLY 287-A, GLU 167-C, GLY 163-C
H-bond	2 ×	-	4 ×	2 ×	3 × BZD:H34 - VAL 159-A:O LEU 162-B:HN - BZD:O2 LEU 162-C:HN - BZD:O1	4 × TRP 106-B:HN - GNST:O4 TYR 268-A:HH - GNST:O2 GNST:H10 - GLY 266-A:O ALA 288-B:HN - GNST:O4	3 × RLX:H27 - ASP 286-A:O LYS 105-A:HZ2 - RLX:O2 ALA 288-A:HN - RLX:O4

Abbreviations: BZD = bazedoxifene; GNST = genistin; RLX = raloxifene

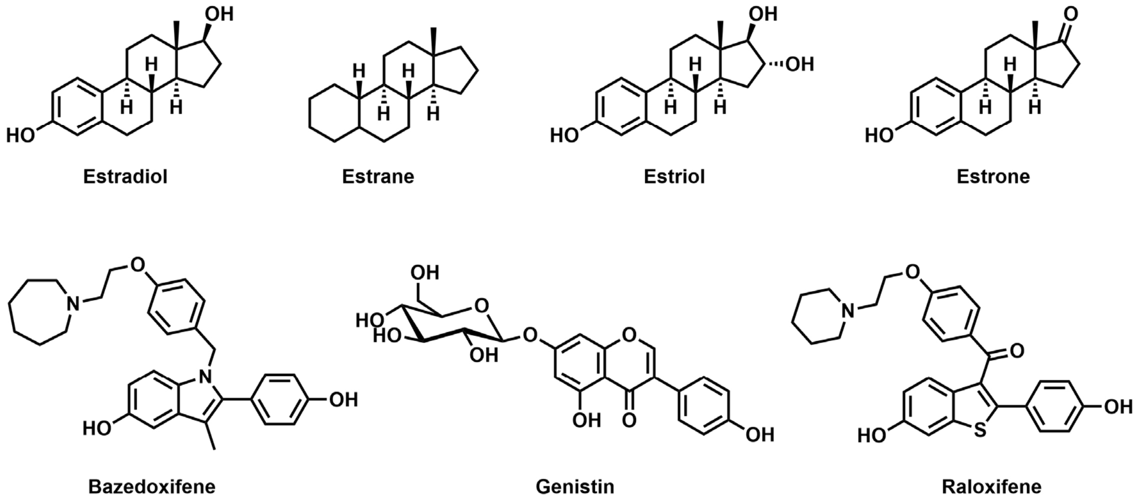

Figure S1. Structures of ERMs used for docking studies

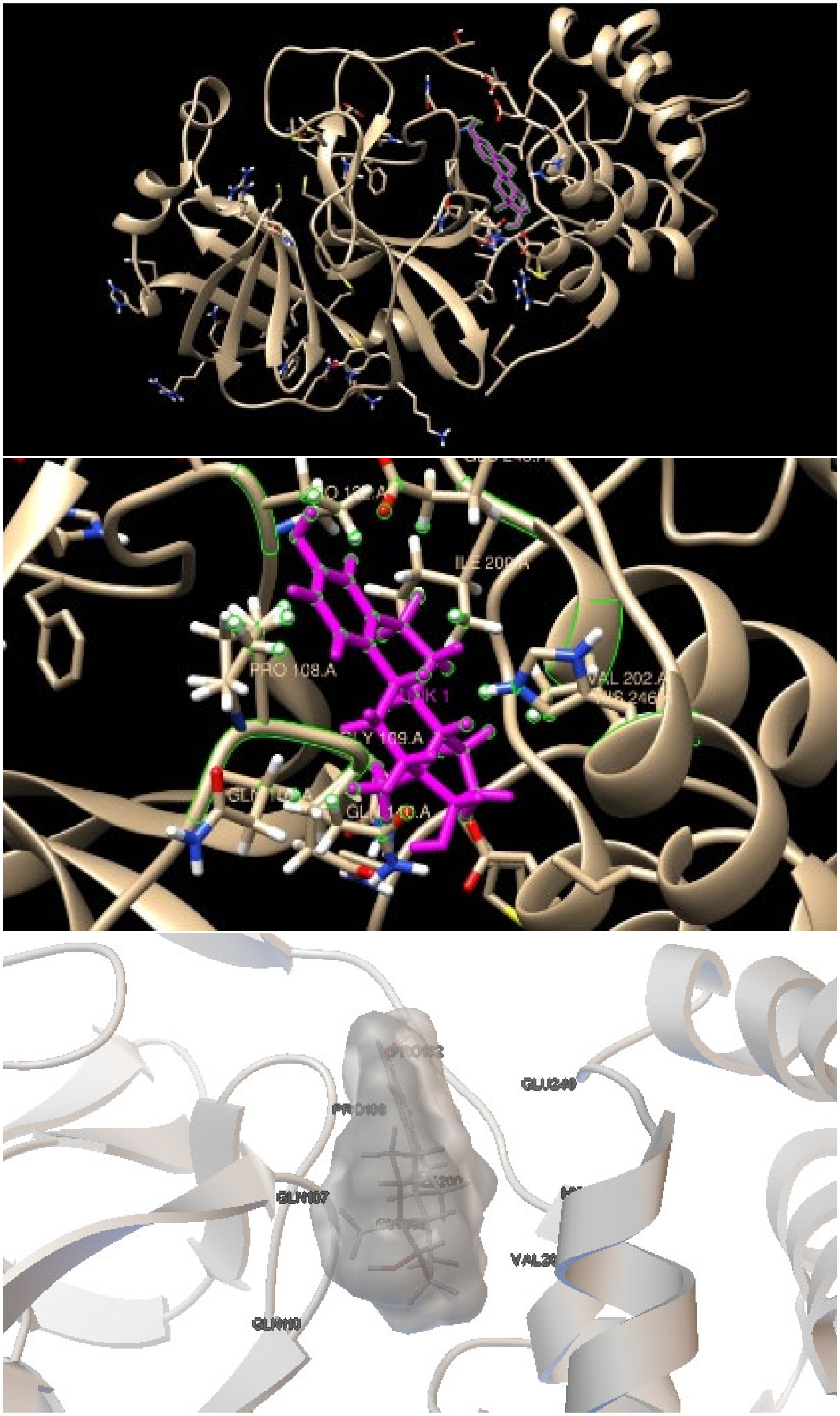

Figure S2. The best docking pose of estradiol to the SARS-CoV-2 main protease (M^{pro})

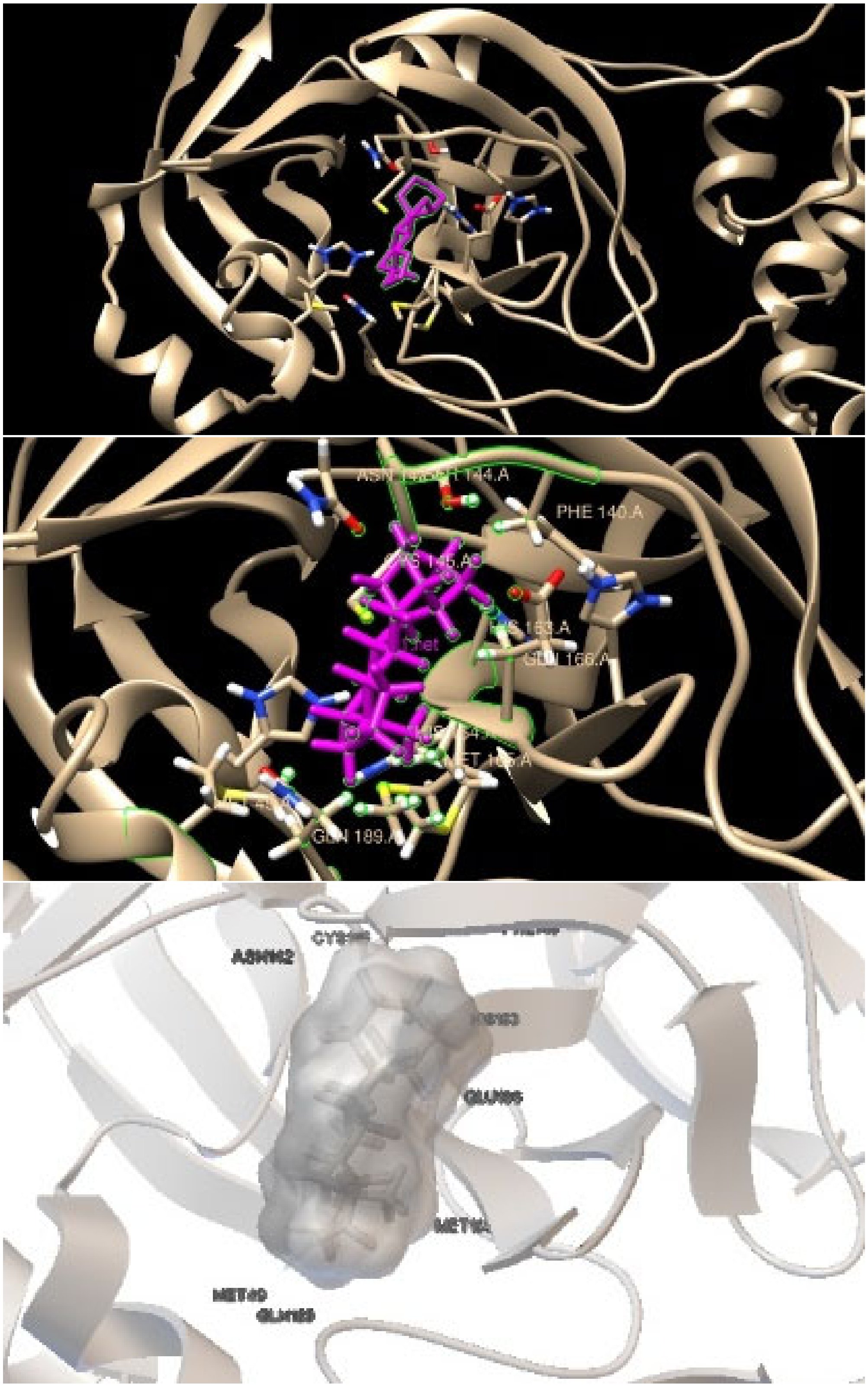

Figure S3. The best docking pose of estrane to the SARS-CoV-2 main protease (M^{pro})

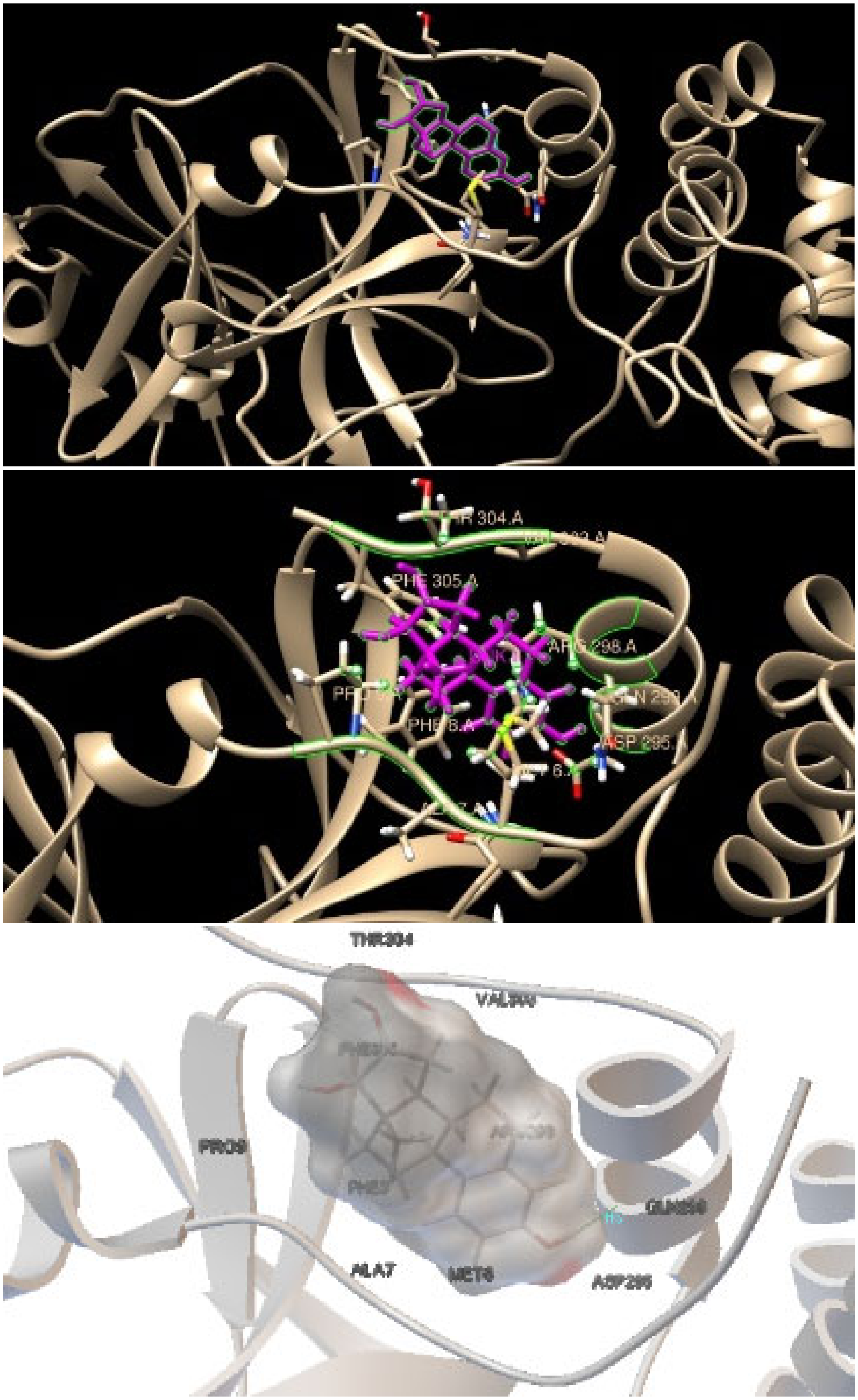

Figure S4. The best docking pose of estriol to the SARS-CoV-2 main protease (M^{pro})

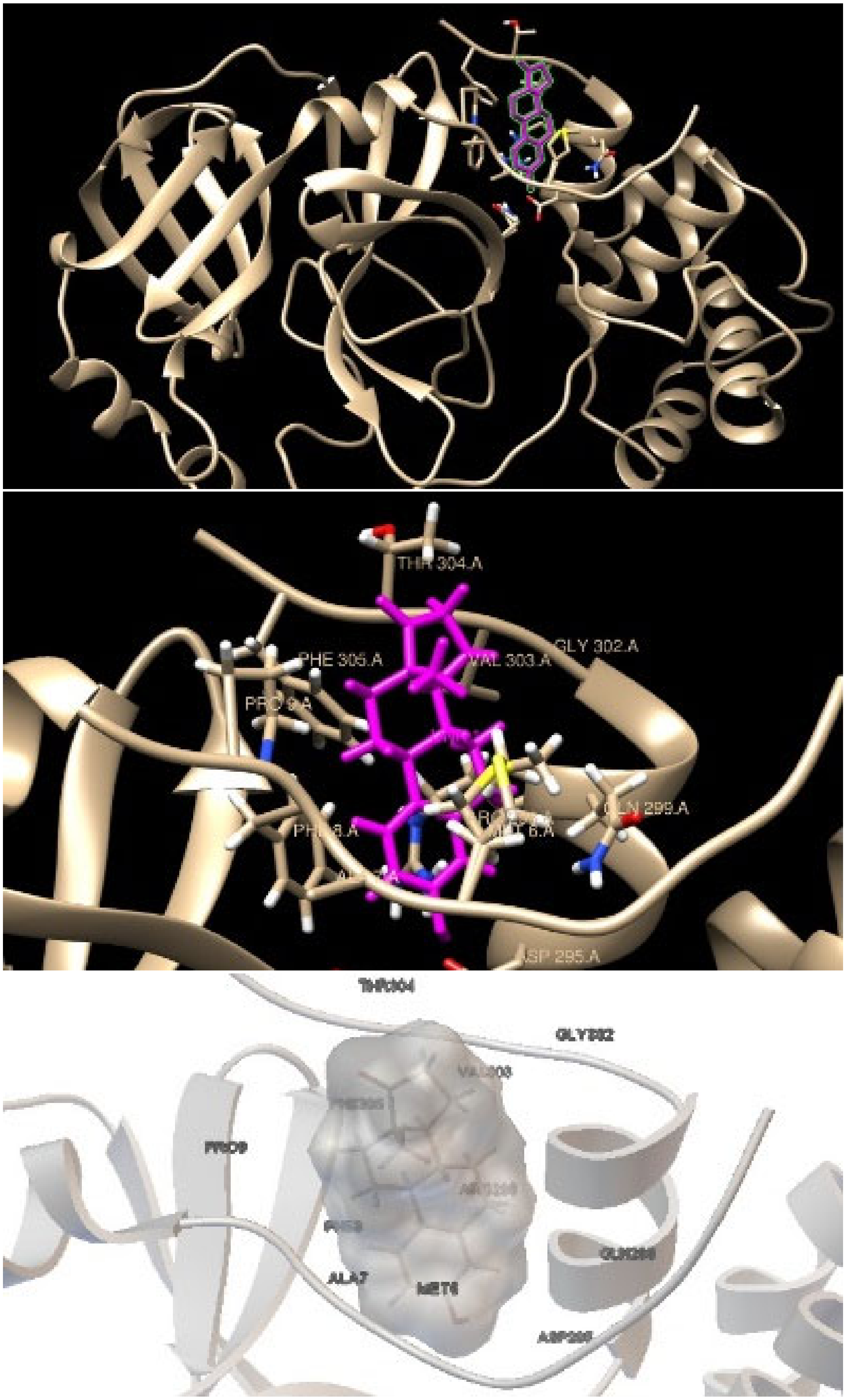

Figure S5. The best docking pose of estrone to the SARS-CoV-2 main protease (M^{pro})

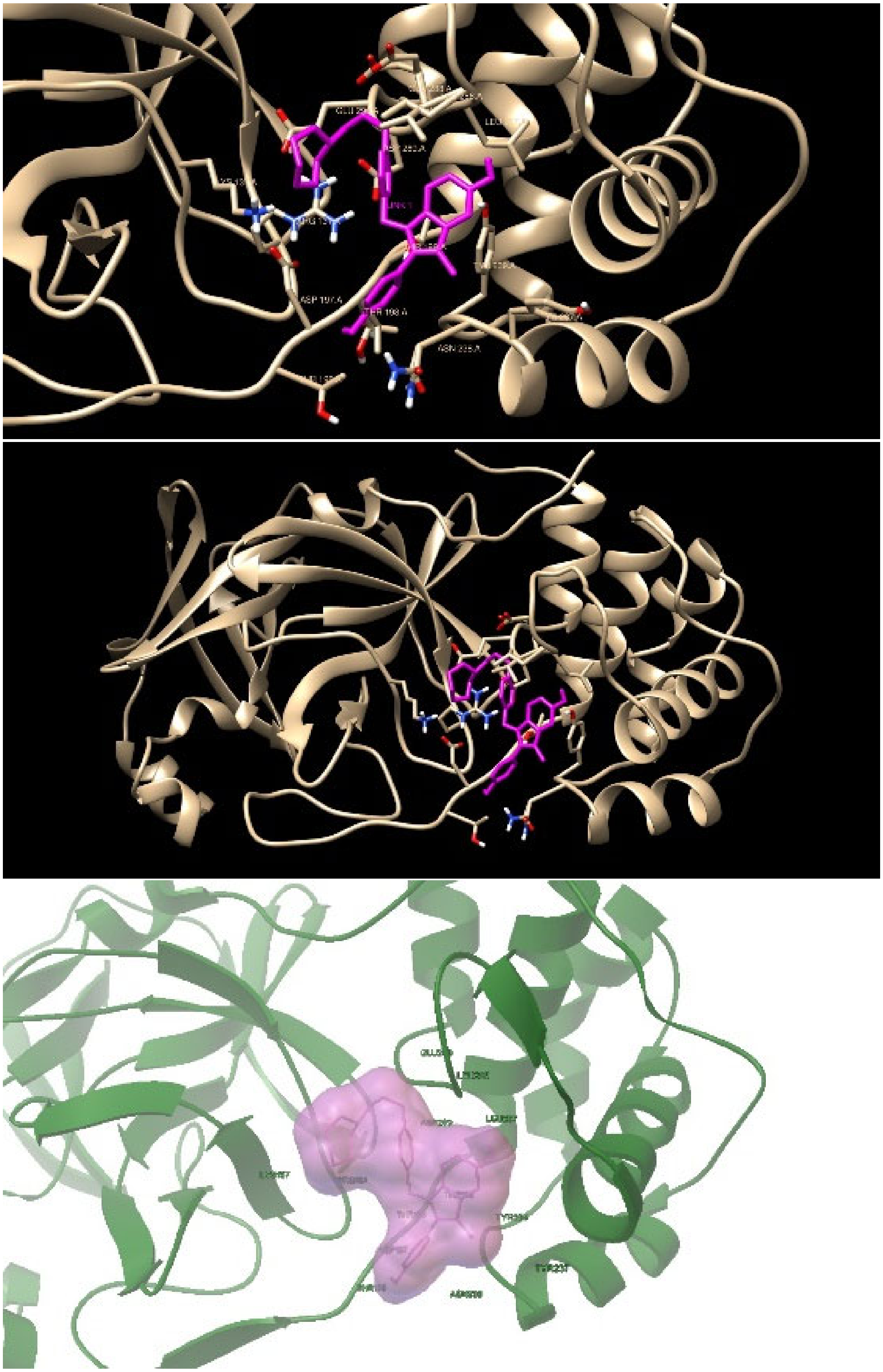

Figure S6. The best docking pose of bazedoxifen to the SARS-CoV-2 main protease (M^{pro})

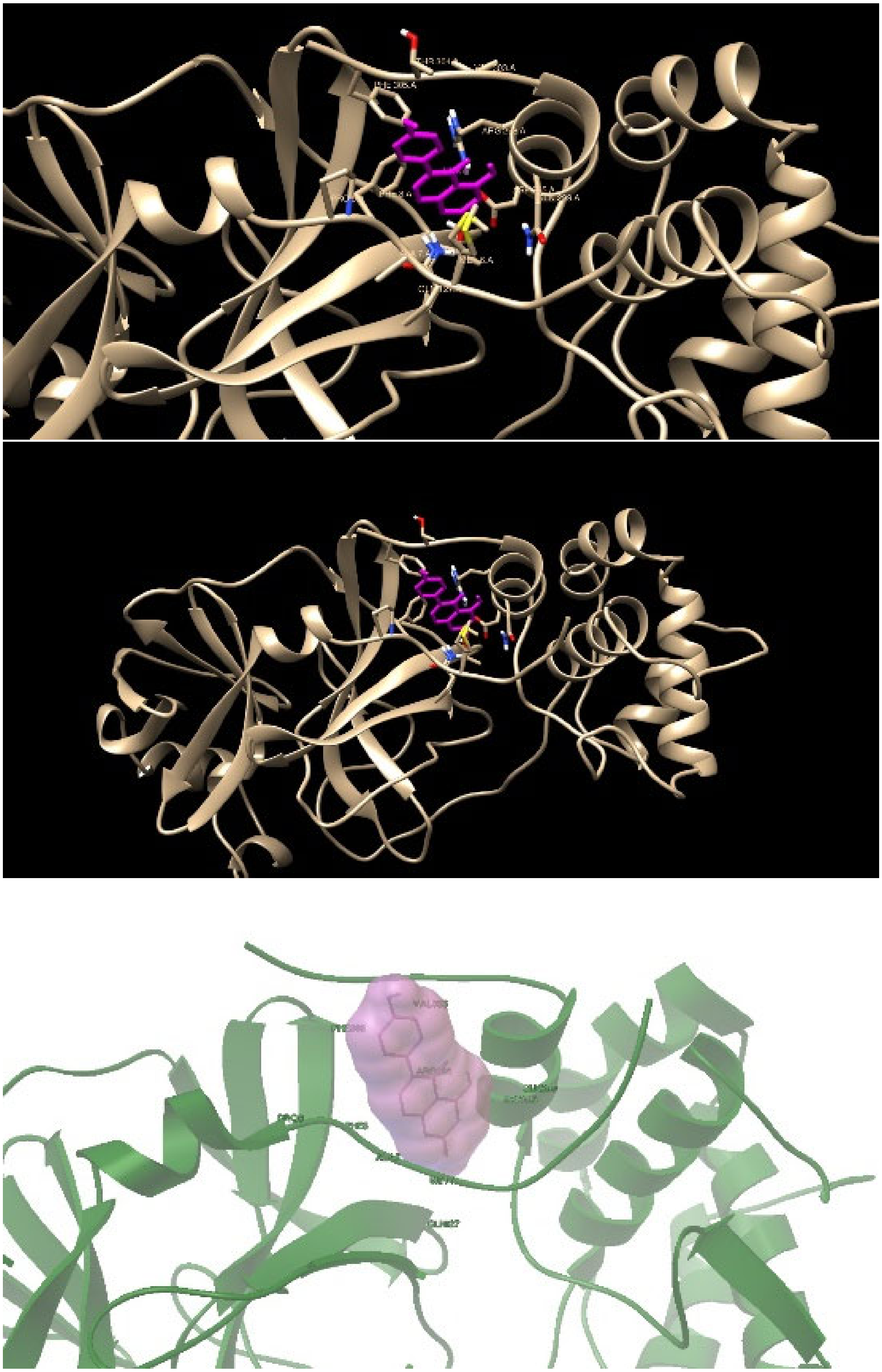

Figure S7. The best docking pose of genistin to the SARS-CoV-2 main protease (M^{pro})

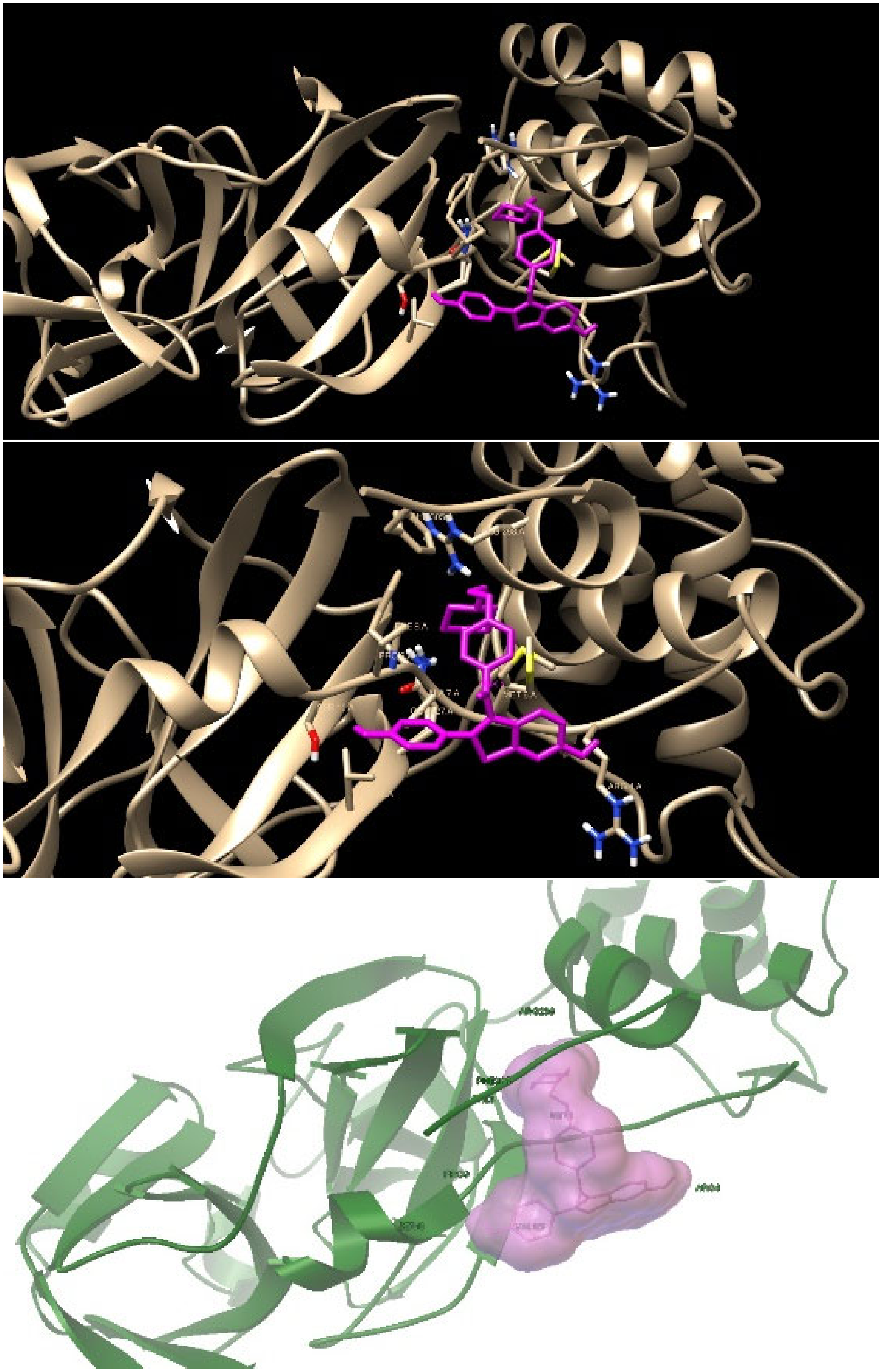

Figure S8. The best docking pose of raloxifene to the SARS-CoV-2 main protease (M^{pro})

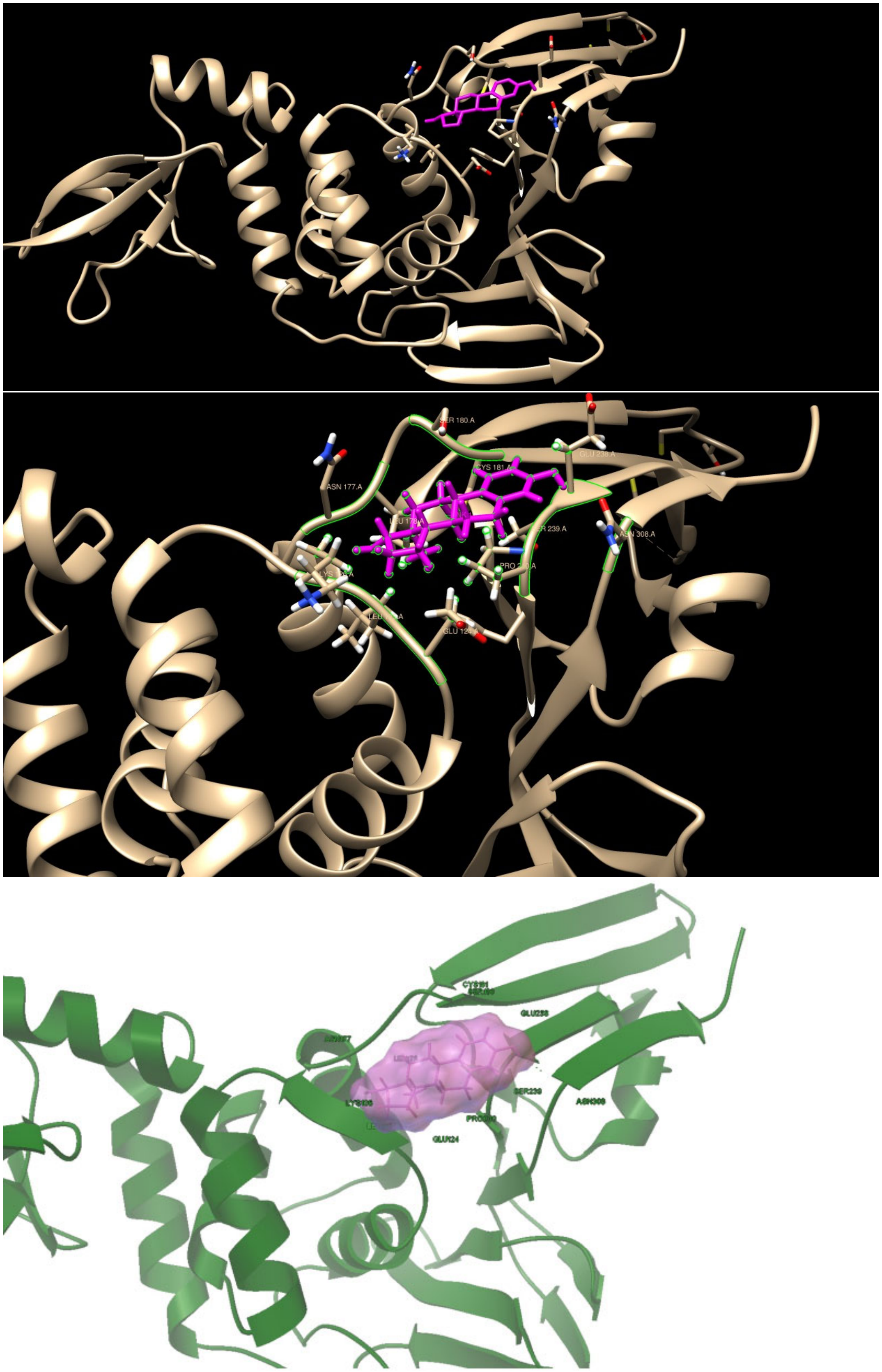

Figure S9. The best docking pose of estradiol to the SARS-CoV-2 papain-like protease (mono PL^{pro})

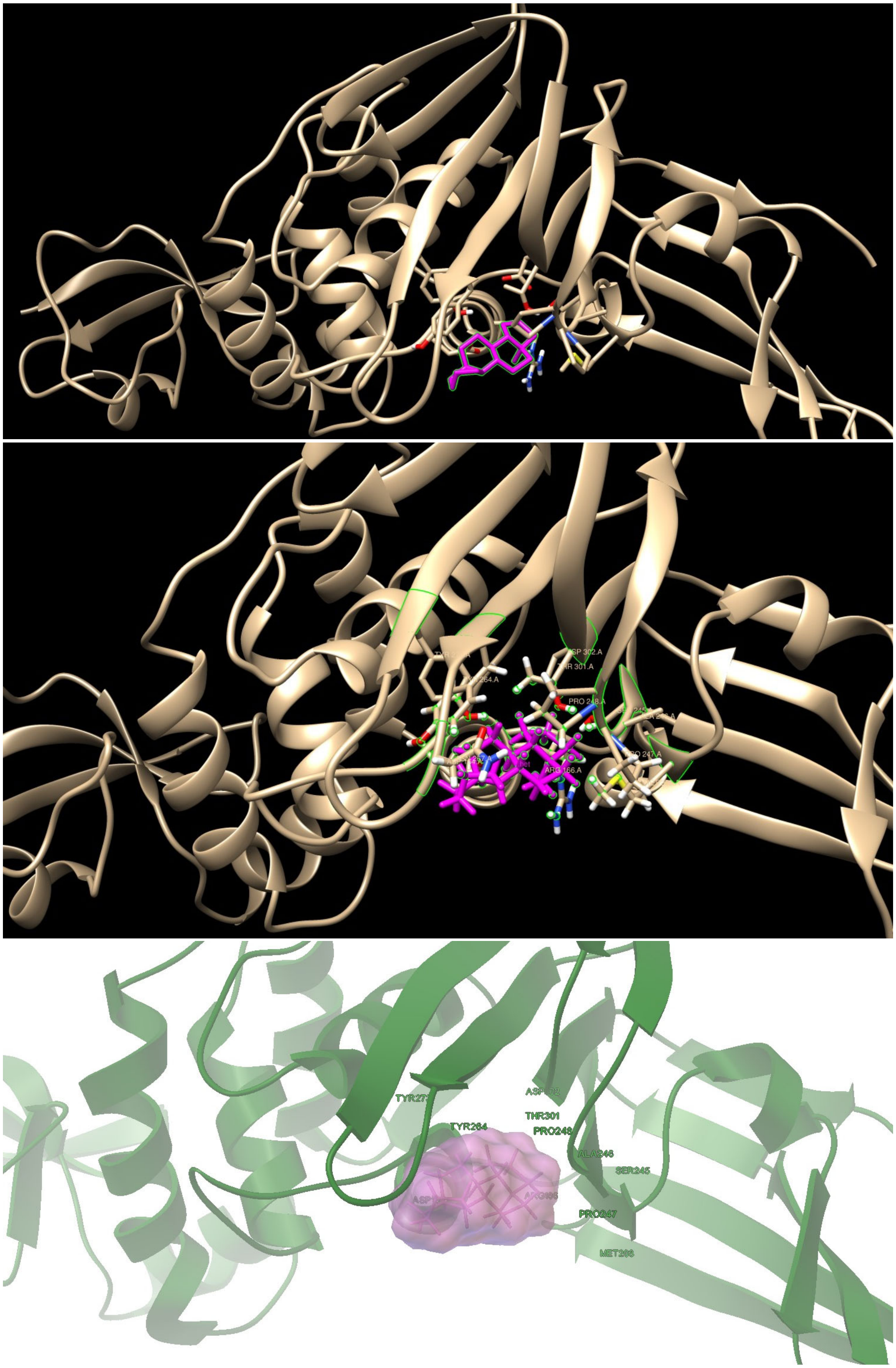

Figure S10. The best docking pose of estrane to the SARS-CoV-2 papain-like protease (mono PL^{pro})

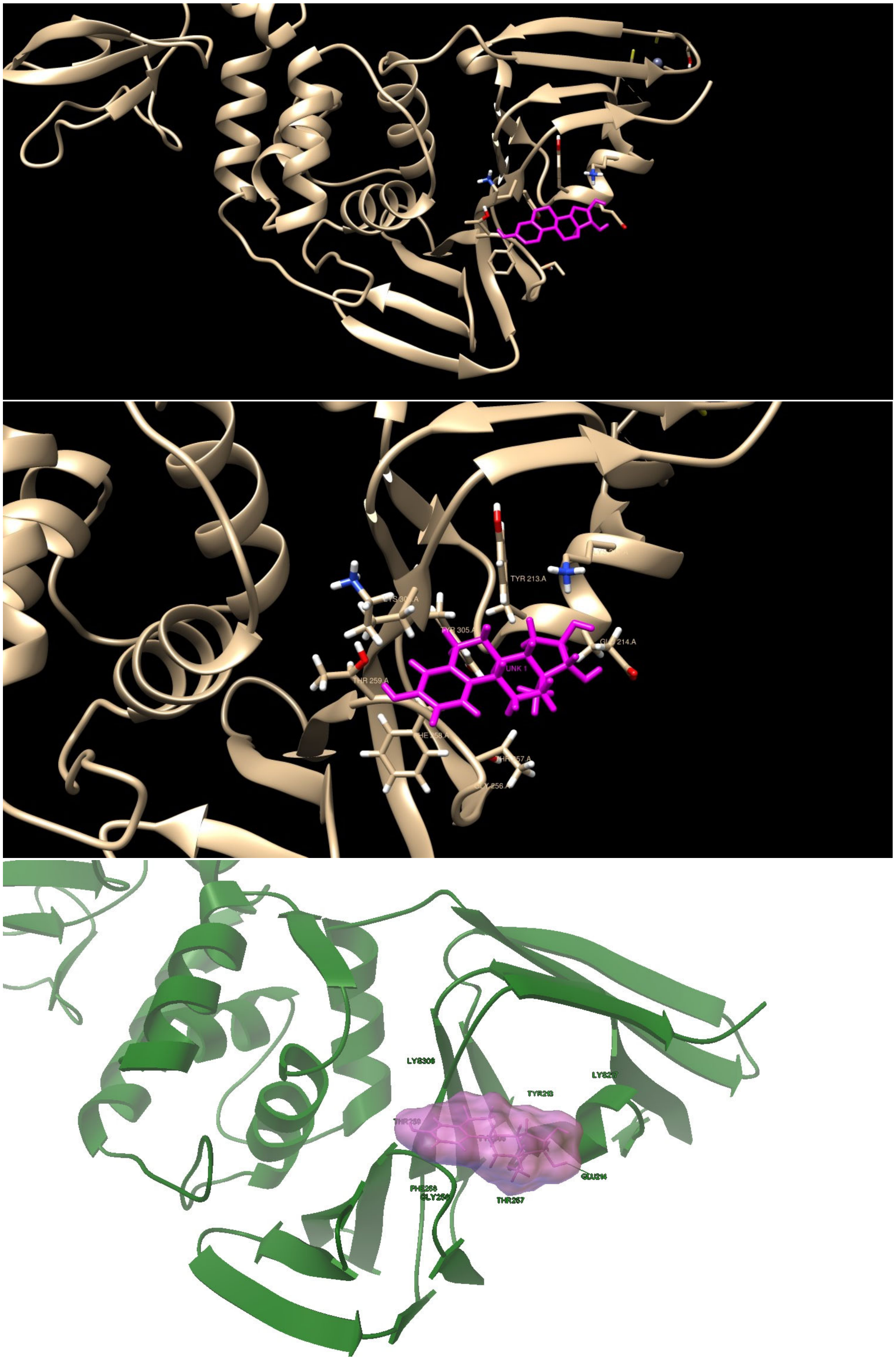

Figure S11. The best docking pose of estriol to the SARS-CoV-2 papain-like protease (mono PL^{pro})

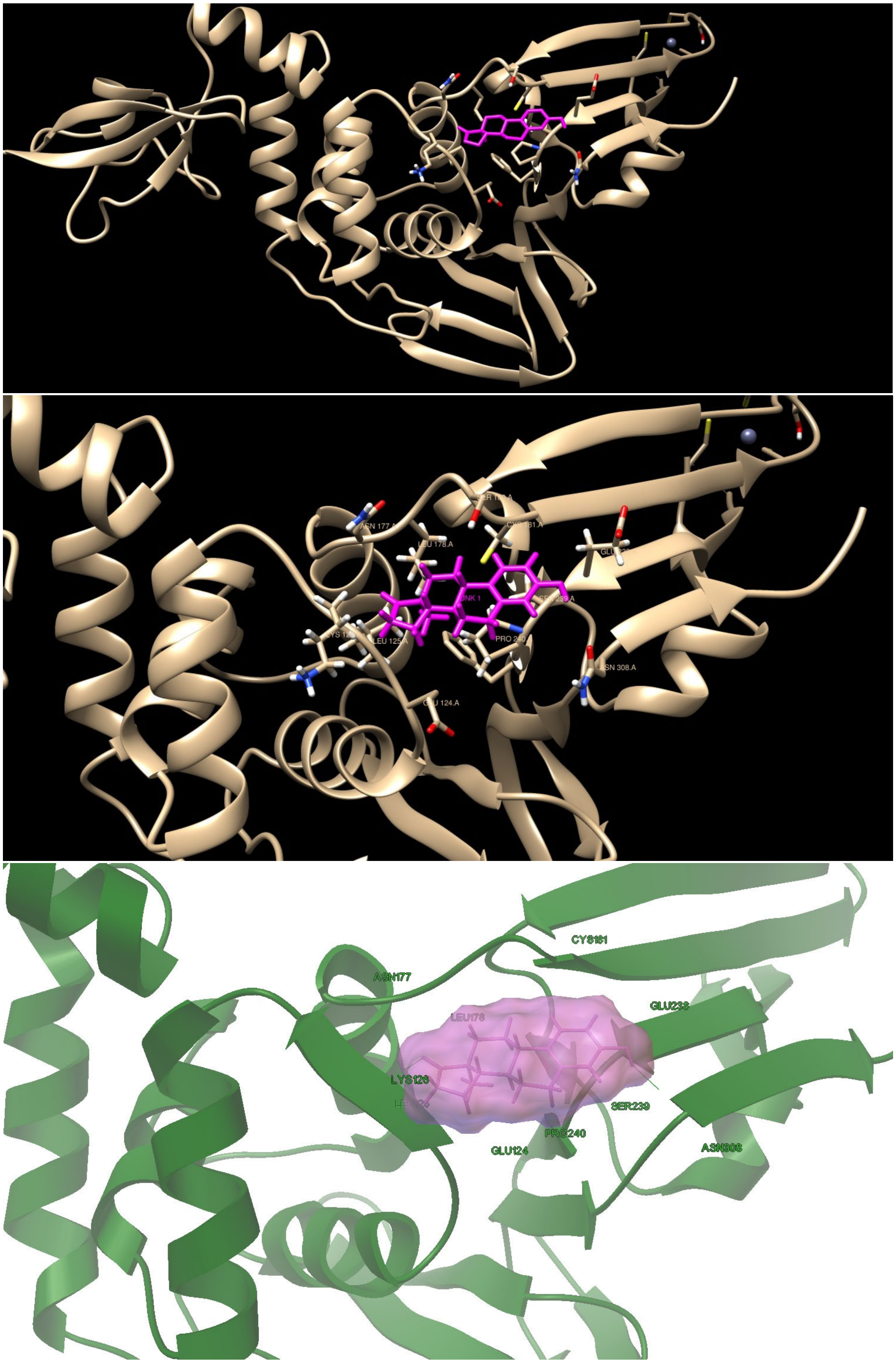

Figure S12. The best docking pose of estrone to the SARS-CoV-2 papain-like protease (mono PL^{pro})

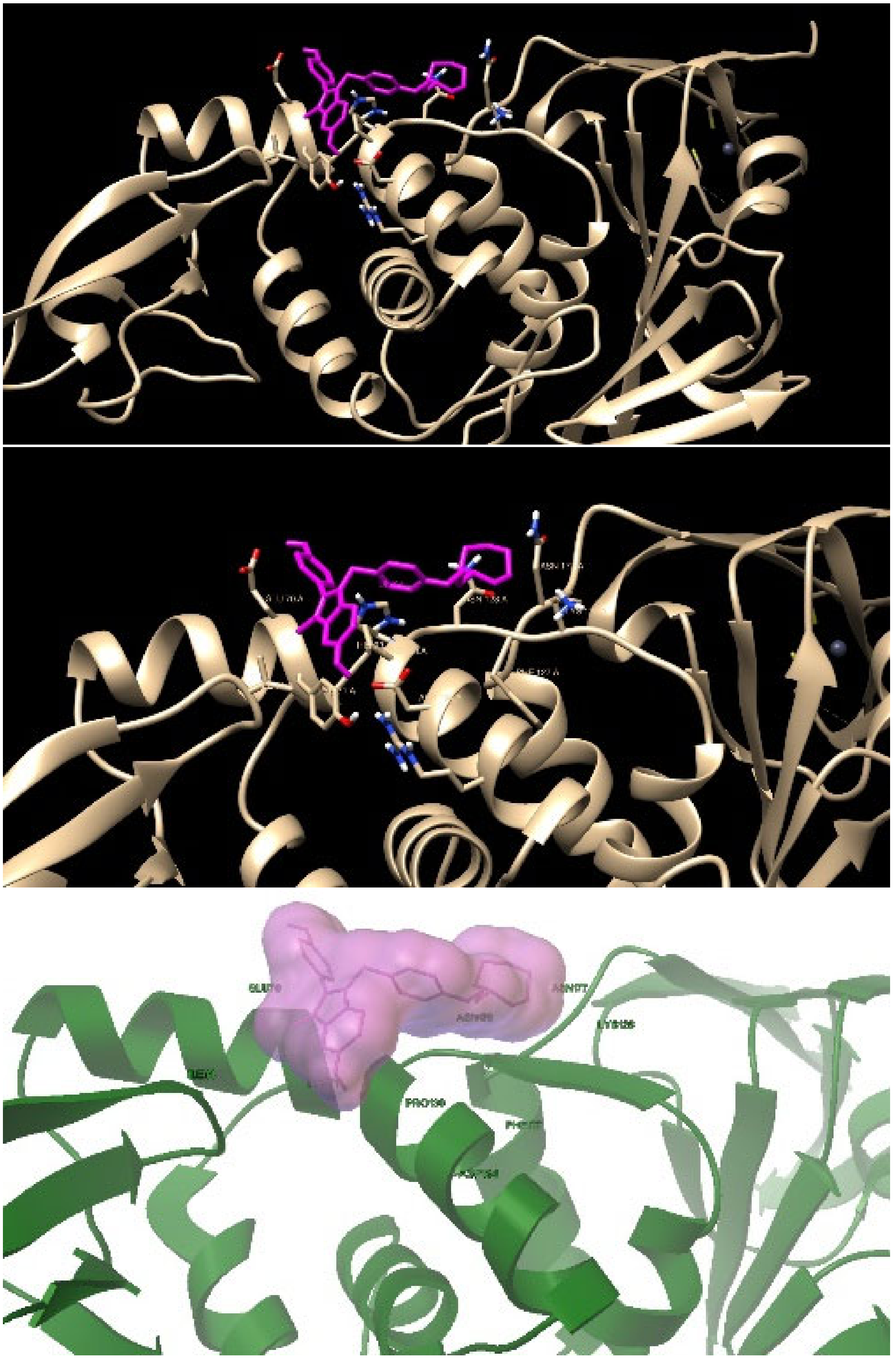

Figure S13. The best docking pose of bazedoxifene to the SARS-CoV-2 papain-like protease (mono PL^{pro})

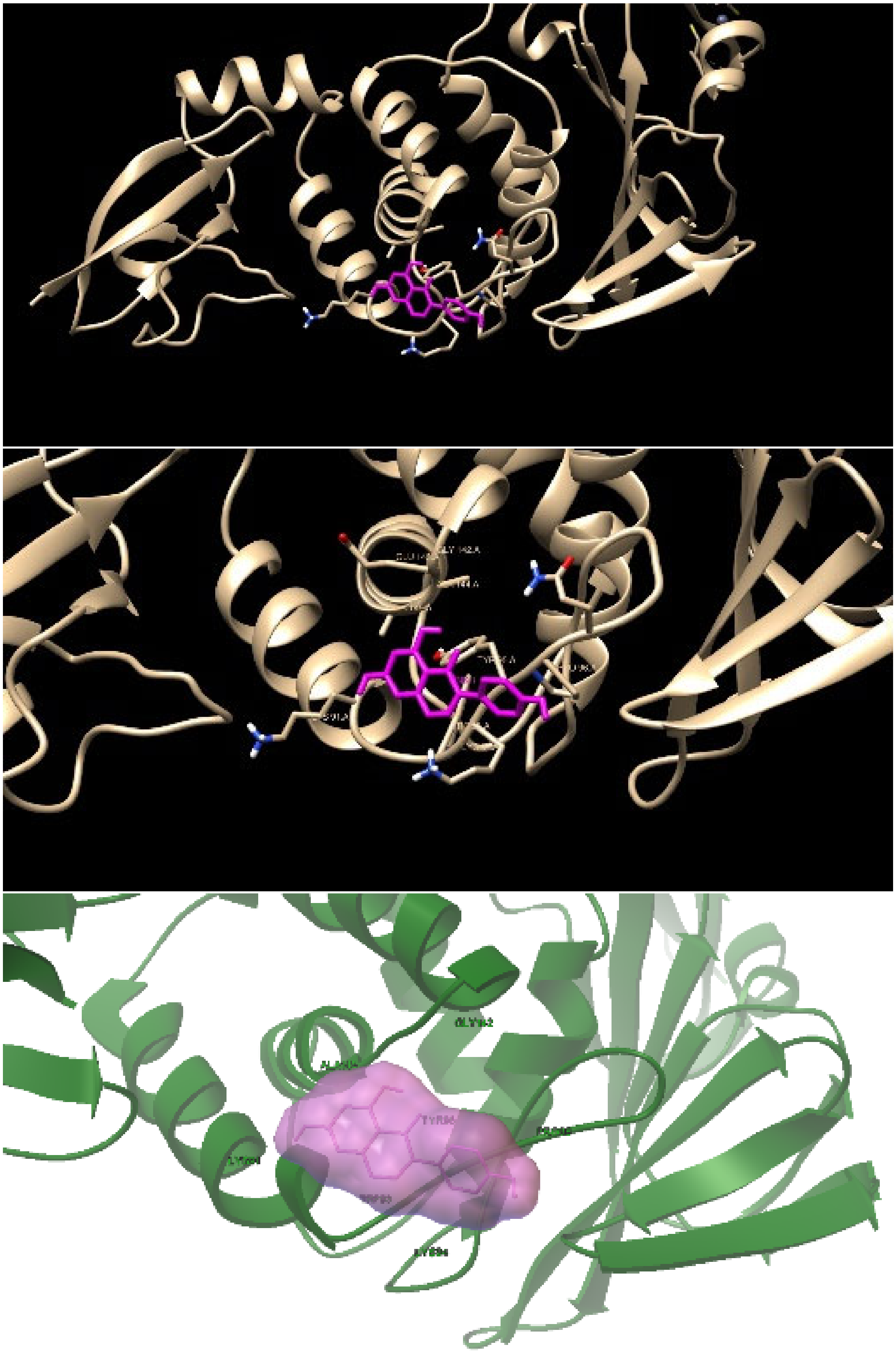

Figure S14. The best docking pose of genistin to the SARS-CoV-2 papain-like protease (mono PL^{pro})

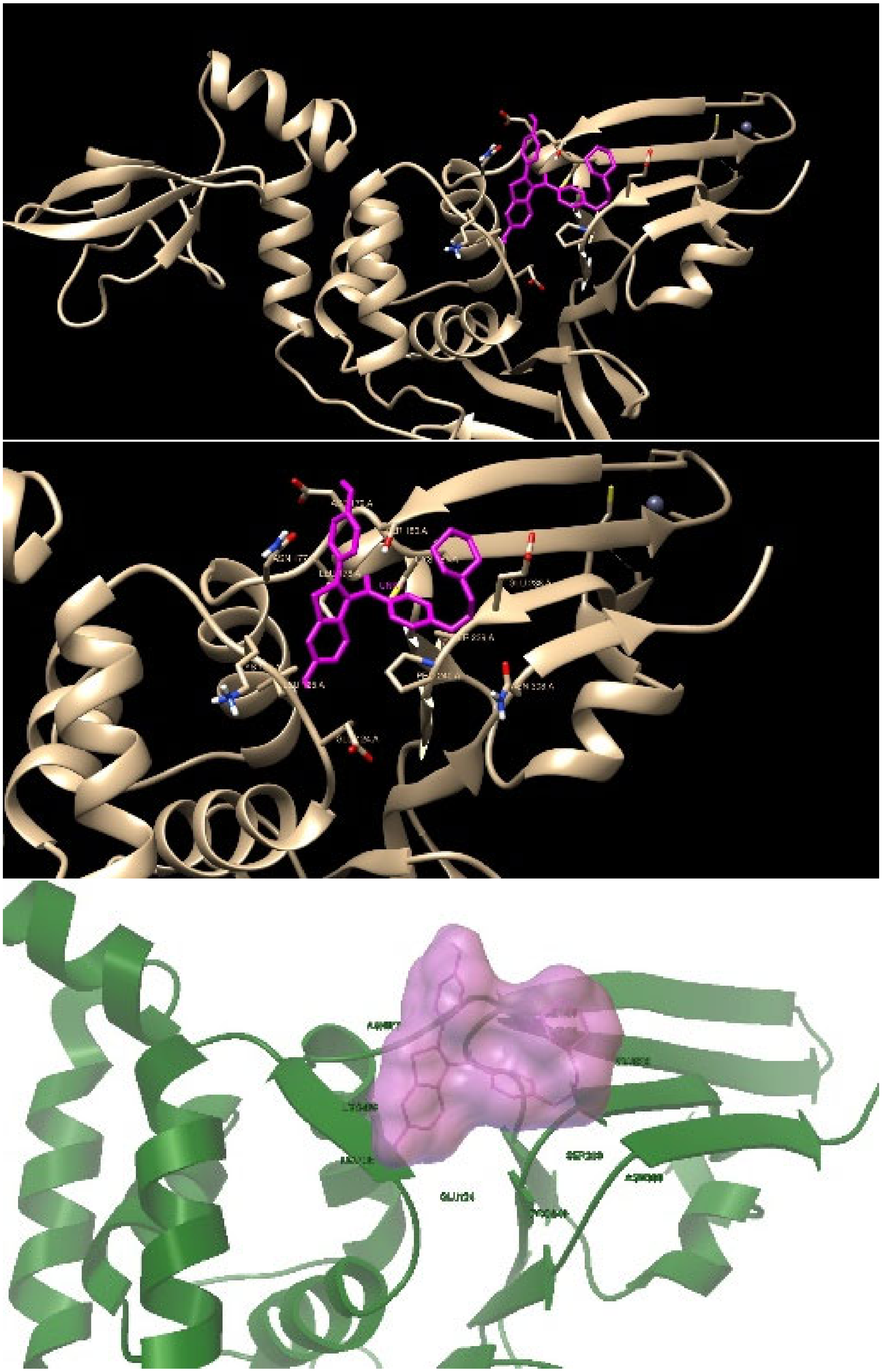

Figure S15. The best docking pose of raloxifene to the SARS-CoV-2 papain-like protease (mono PL^{pro})

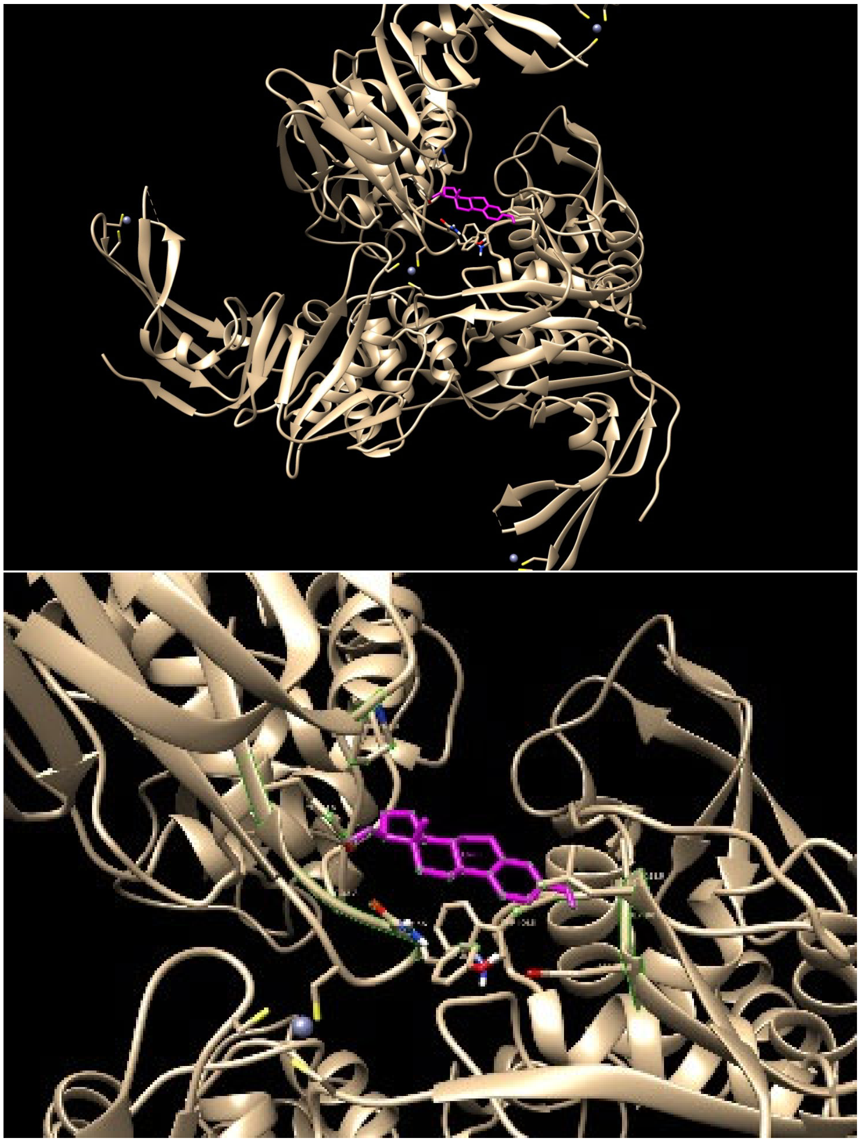

Figure S16. The best docking pose of estradiol to the SARS-CoV-2 papain-like protease (trimer PL^{pro})

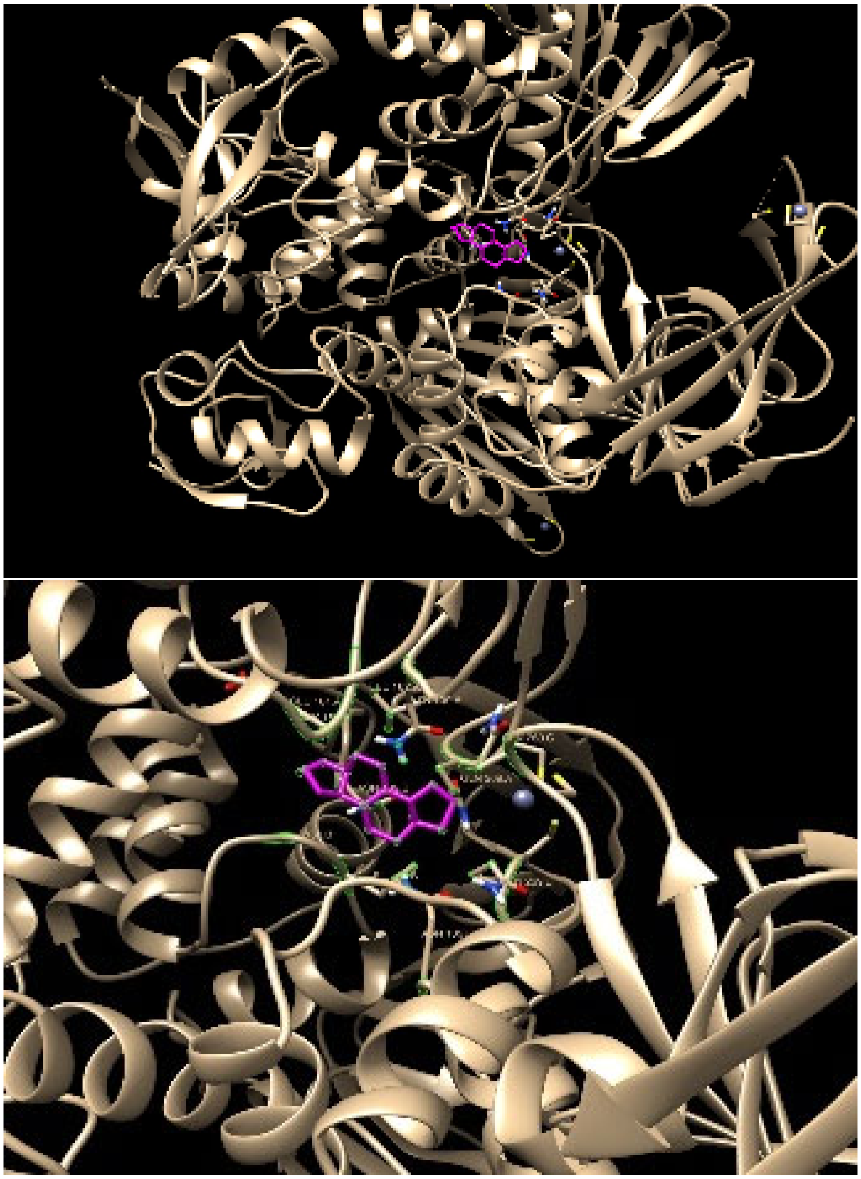

Figure S17. The best docking pose of estrane to the SARS-CoV-2 papain-like protease (trimer PL^{pro})

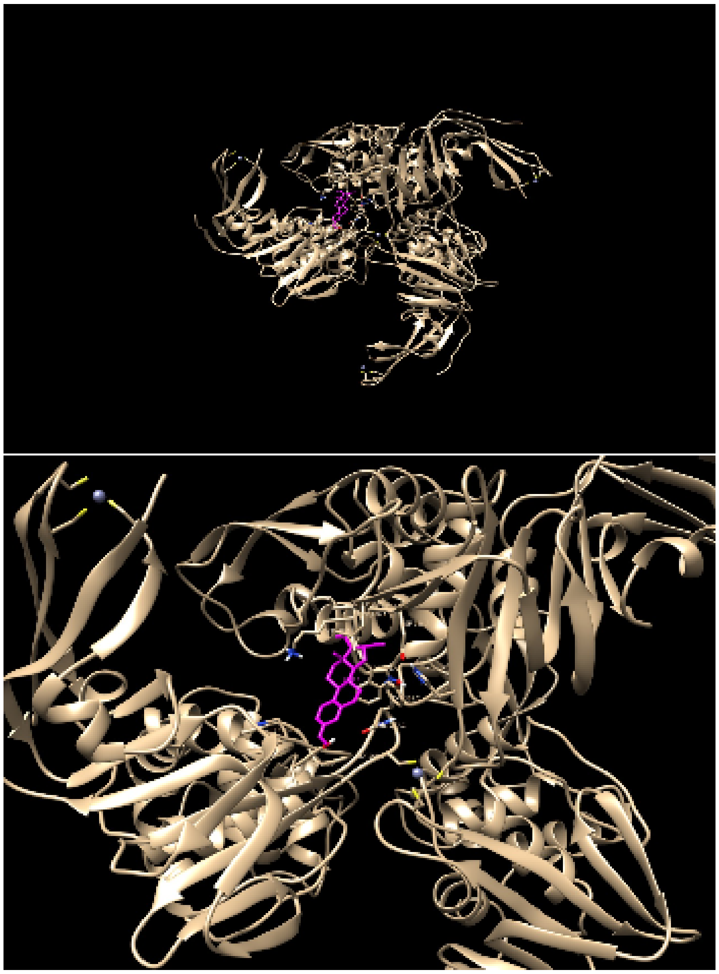

Figure S18. The best docking pose of estriol to the SARS-CoV-2 papain-like protease (trimer PL^{pro})

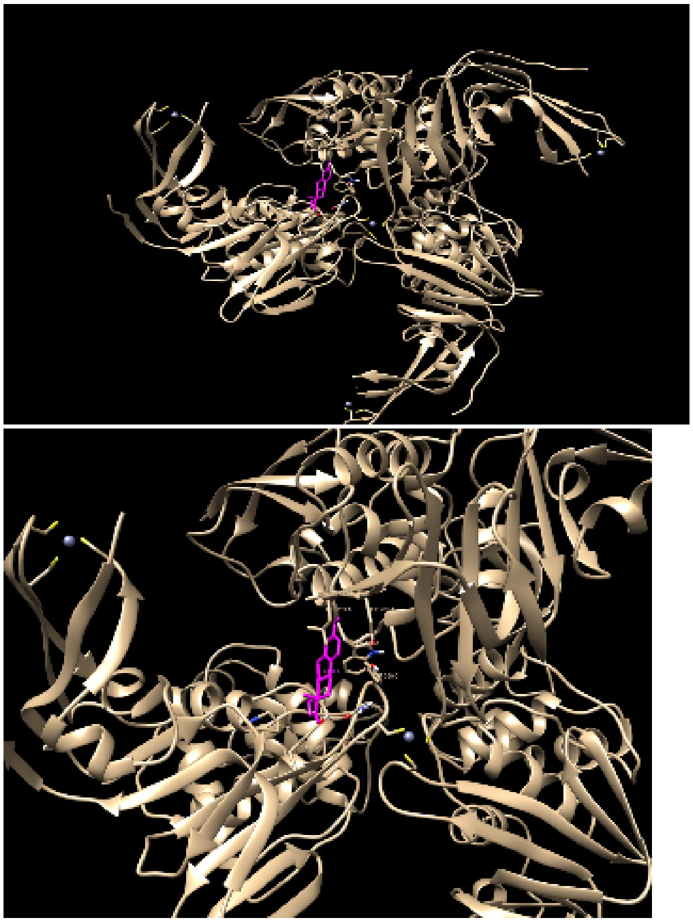

Figure S19. The best docking pose of estrone to the SARS-CoV-2 papain-like protease (trimer PL^{pro})

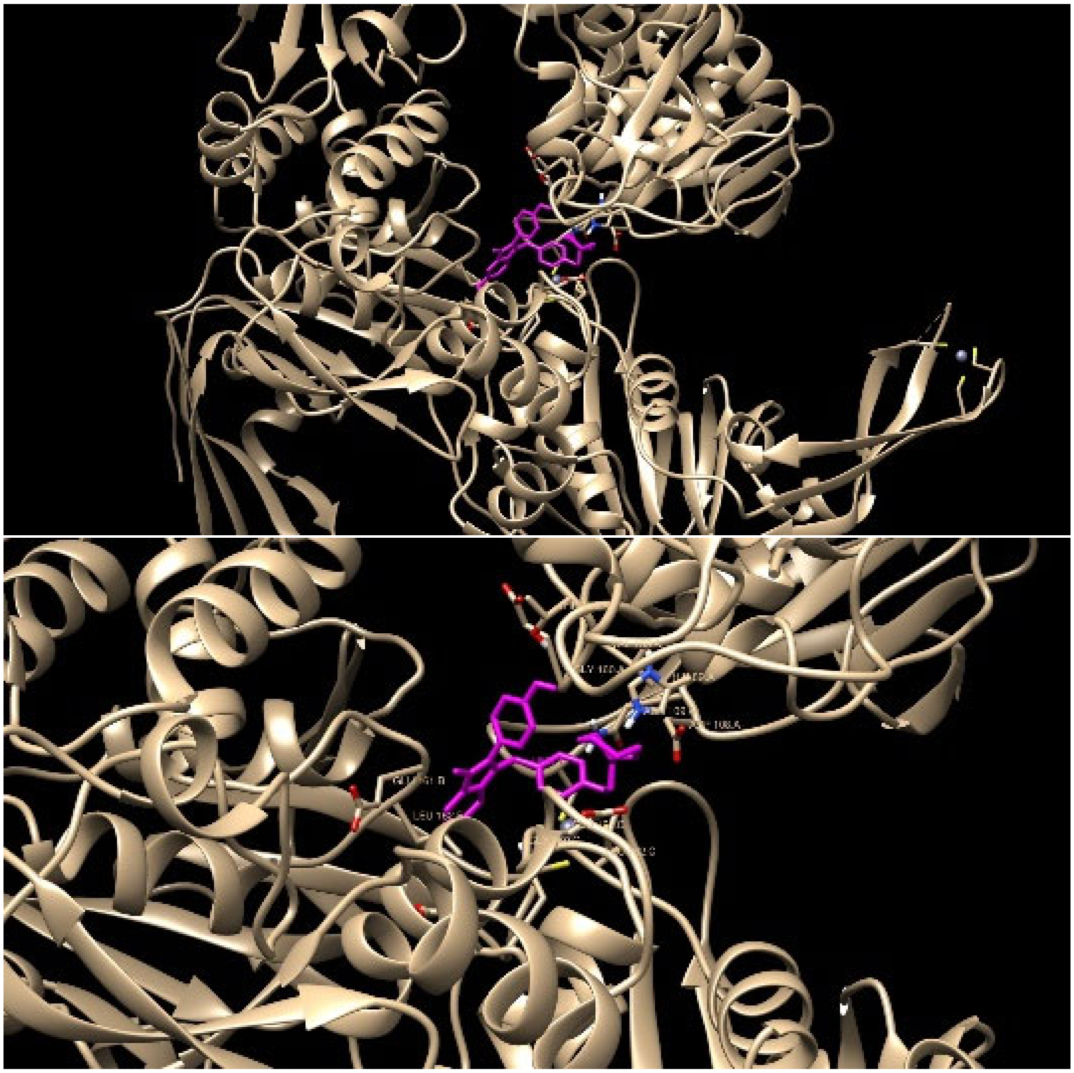

Figure S20. The best docking pose of bazedoxifene to the SARS-CoV-2 papain-like protease (trimer PL^{pro})

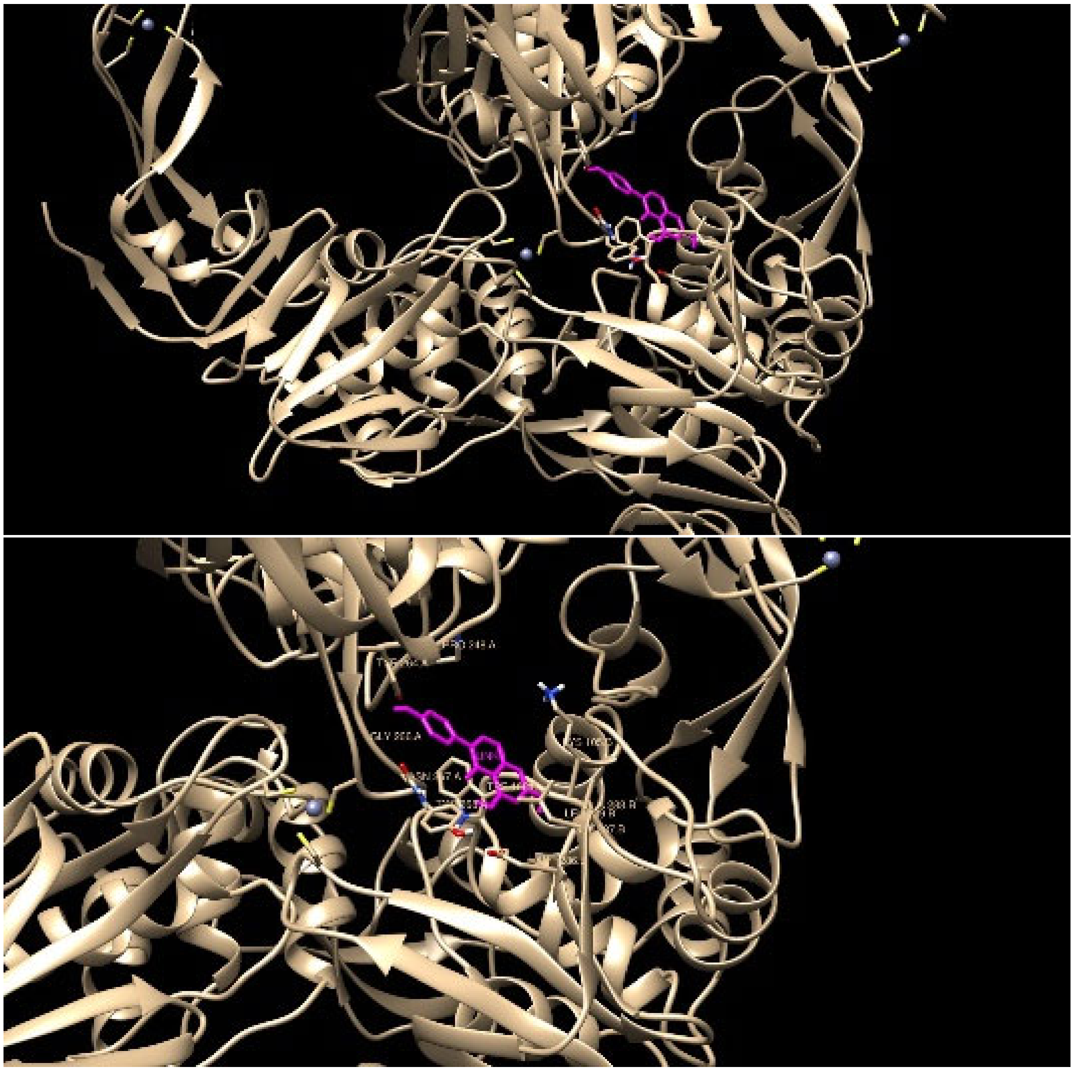

Figure S21. The best docking pose of genistin to the SARS-CoV-2 papain-like protease (trimer PL^{pro})

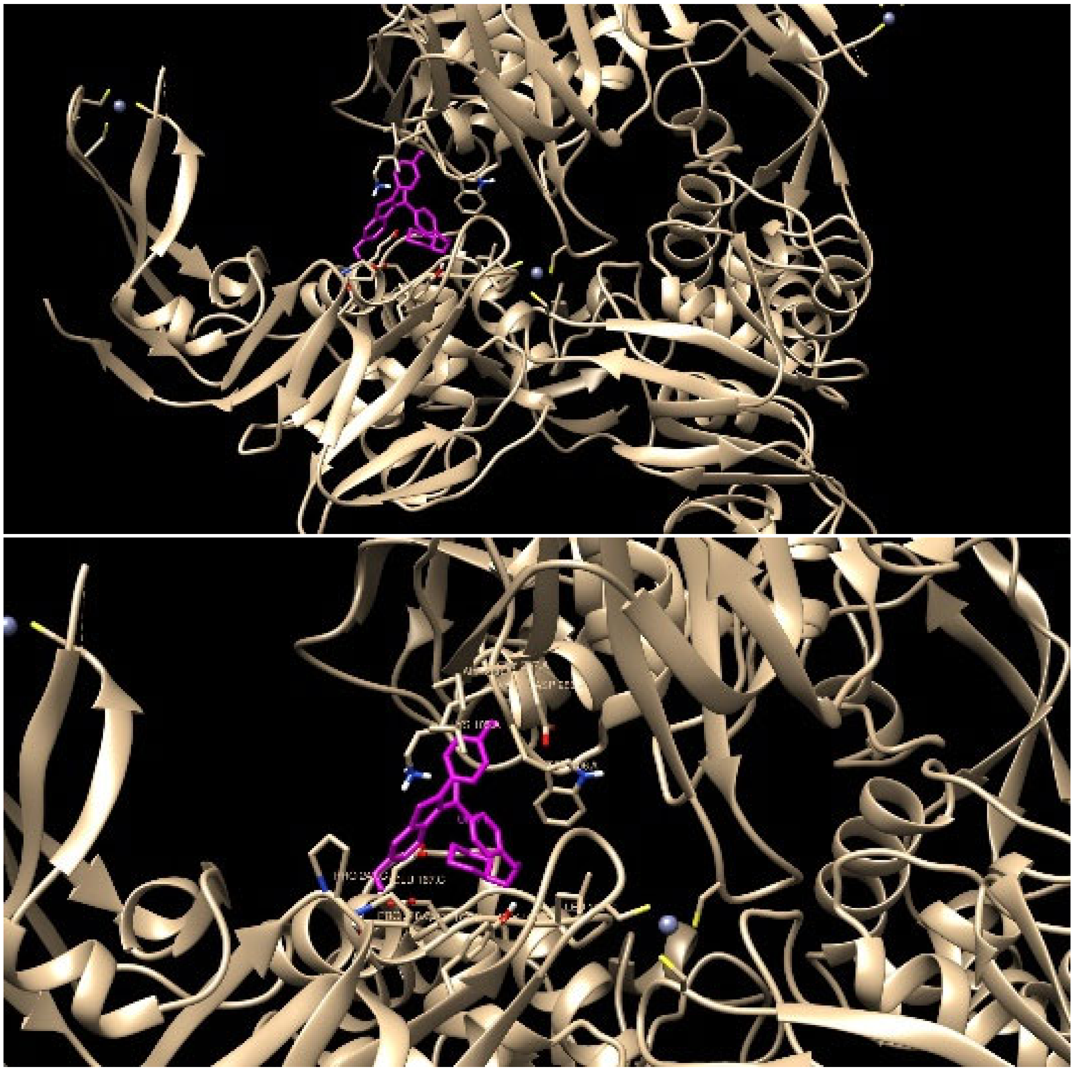

Figure S22. The best docking pose of raloxifene to the SARS-CoV-2 papain-like protease (trimer PL^{pro})
